# Phosphorylation Dynamics in a flg22-Induced, G Protein–Dependent Network Reveals the AtRGS1 Phosphatase

**DOI:** 10.1016/j.mcpro.2023.100705

**Published:** 2023-12-20

**Authors:** Justin M. Watkins, Christian Montes, Natalie M. Clark, Gaoyuan Song, Celio Cabral Oliveira, Bharat Mishra, Libuse Brachova, Clara M. Seifert, Malek S. Mitchell, Jing Yang, Pedro Augusto Braga dos Reis, Daisuke Urano, M. Shahid Muktar, Justin W. Walley, Alan M. Jones

**Affiliations:** 1 Department of Biology, University of North Carolina at Chapel Hill, Chapel Hill, North Carolina, USA; 2 Department of Plant Pathology and Microbiology, Iowa State University, Ames, Iowa, USA; 3 Department of Biochemistry and Molecular Biology/BIOAGRO, Universidade Federal de Viçosa, Viçosa, Brazil; 4 Department of Biology, University of Alabama-Birmingham, Birmingham, Alabama, USA; 5 Department of Biochemistry, Biophysics and Molecular Biology, Iowa State University, Ames, Iowa, USA; 6 Department of Pharmacology, University of North Carolina at Chapel Hill, Chapel Hill, North Carolina, USA

**Keywords:** atbβ, substrate recognition subunit of Ser/Thr PP2A phosphatase, AtRGS1, *Arabidopsis thaliana* regulator of G signaling 1 protein, flg22, 22-amino peptide released from bacterial flagellin, MAMP, Microbe-associated molecular pattern

## Abstract

The microbe-associated molecular pattern flg22 is recognized in a flagellin-sensitive 2–dependent manner in root tip cells. Here, we show a rapid and massive change in protein abundance and phosphorylation state of the Arabidopsis root cell proteome in WT and a mutant deficient in heterotrimeric G-protein–coupled signaling. flg22-induced changes fall on proteins comprising a subset of this proteome, the heterotrimeric G protein interactome, and on highly-populated hubs of the immunity network. Approximately 95% of the phosphorylation changes in the heterotrimeric G-protein interactome depend, at least partially, on a functional G protein complex. One member of this interactome is ATBα, a substrate-recognition subunit of a protein phosphatase 2A complex and an interactor to *Arabidopsis thaliana* Regulator of G Signaling 1 protein (AtRGS1), a flg22-phosphorylated, 7-transmembrane spanning modulator of the nucleotide-binding state of the core G-protein complex. A null mutation of *ATB*α strongly increases basal endocytosis of AtRGS1. AtRGS1 steady-state protein level is lower in the *atbα* mutant in a proteasome-dependent manner. We propose that phosphorylation-dependent endocytosis of AtRGS1 is part of the mechanism to degrade AtRGS1, thus sustaining activation of the heterotrimeric G protein complex required for the regulation of system dynamics in innate immunity. The PP2A(ATBα) complex is a critical regulator of this signaling pathway.

## Highlights

• The bacterial MAMP flgs22 induces reactive oxygen production in roots.
• flg22 elicits a profound change in the root phosphoproteome.
• This discovery approach revealed a missing phosphatase to a G protein component.
• This phosphatase regulates the phospho-barcode on Regulator of G Signaling 1 (RGS1) protein.
• This phospho-barcode instructs RGS1 endocytosis and stability.

## Graphical Abstract

**Figure:**
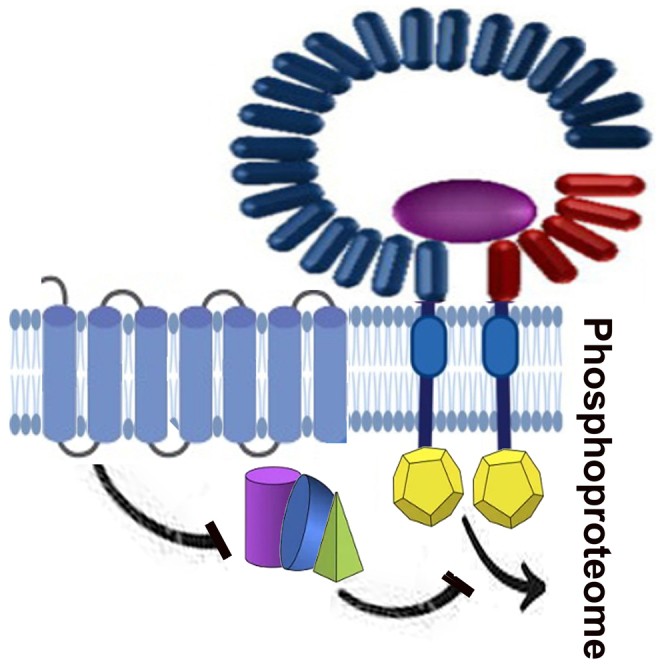

## In Brief

The bacterial MAMP flgs22 induces reactive oxygen production in roots and elicits a profound change in the root phosphoproteome in a manner that is profoundly dependent on an intact heterotrimeric G protein that is itself regulated by a 7-transmembrane Regulator of G Signaling (RGS) protein. flg22 induces phosphorylation of this RGS protein and consequently its degradation. The phosphatase that dephosphorylates this RGS to regulate its degradation is here identified as the substrate recognition subunit, ATBα, in a PP2A phosphatase complex.

A coordinated signaling relay begins with the perception of external signals by membrane-bound receptors, such as G-protein coupled receptors (GPCRs) in animal cells. Similar to animals, the coupler of the activated receptor and cytoplasmic responses is the heterotrimeric G protein complex in Arabidopsis (hereafter, simply G protein complex). The Arabidopsis G protein complex contains one canonical Gα subunit (AtGPA1) or one of three atypical Gα subunits, XLGs (1), Gβ subunit (AGB1), one of three Gγ subunits (AGG1, AGG2, and AGG3) (2, 3). However, the plant G protein releases GDP, binds GTP, and undergoes a conformational change to be active without GPCRs; rather, the activation status is modulated, in part, by regulator of G signaling 1 (AtRGS1 in Arabidopsis), another component of the G-protein complex (4, 5). AtRGS1 contains a 7TM domain, an RGS domain that catalyzes the intrinsic GTP hydrolysis activity of Gα (5, 6, 7, 8), and a C-terminal cluster of phosphorylation sites (9, 10, 11). AtGPA1 is phosphorylated thus altering function (12). In addition, derepression occurs when AtRGS1 internalizes away from AtGPA1 upon signal perception, and this endocytosis depends on the phosphorylation of a phosphocluster in the C-terminal tail. The kinases that phosphorylate AtRGS1 include with no lysine (WNK) kinases and BAK1 (10, 13). BAK1 is a transient component of the flg22 receptor, FLS2 (14). flg22 is a microbe-associated molecular patter (MAMP) that sets into motion many aspects of plant immunity. One of these is AtRGS1 endocytosis initiated by phosphorylation within the phosphocluster (9, 15, 16, 17, 18). While various kinases that phosphorylate AtRGS1 and G protein subunits have been identified, to date, the phosphatase in this phosphorylation-dephosphorylation cycle is unknown.

The Arabidopsis G protein interactome project identified over 400 direct interactions within ∼70 highly interconnected core components (19). Those interactomes combined with further characterizations with biochemical, genetic, and cell biological evidence revealed new regulators downstream of the G protein complex such as the WNK kinases. Through *in vitro* screening of 70 receptor kinases, we also showed that BAK1 directly phosphorylates AtRGS1 and AtGPA1, as well as their phosphorylation sites, and the structural mechanisms of how their activity is regulated (10, 12, 13, 18, 20). Those large-scale screens revealed direct but static interactions within the plant G protein network that are composed of highly-conserved core nodes (G protein subunits and AtRGS1) throughout eukaryotes, together with peripheral nodes (other regulators and effectors) which largely differ from animals. Advanced proteomics approaches to quantitate posttranslational modifications, such as phosphorylation, can reveal a dynamic signaling flow over time (21). Such time-dependent changes of posttranslational status, combined with a physical interaction map, allowed us to infer direct signaling transmission events between proteins. Two individual phosphorylation targets (AtRGS1 and AtGPA1) are the key molecular signatures controlling G protein activity (22).

Pathogen and MAMP-induced quantitative and system-wide experiments yielded many multidimensional datasets. To better understand the intricate nature of the plant immune signaling cascade, an integrative network was instrumental to decipher the significant players in plant–pathogen interactions (23, 24, 25). For example, network architectural or centrality analyses of global protein–protein interaction networks (*i.e.* interactomes) revealed the preferred pathogenic contact points to hosts (19, 26, 27, 28, 29). With reference to phosphoproteomes, their integration with interactome datasets shows promise for yielding a comprehensive landscape of immune signaling (24, 25). This integration is particularly relevant for plant-pathogen interactomes that encompass nodes as host or pathogen molecules, while edges exhibit interactions as coordinated by the system-wide host responses. These interactions highlight novel players that induce immune or defense responses under pathogen infection (25).

Using quantitative proteomics, we report flg22-dependent remodeling of the phosphoproteome that highlights G protein–dependent phosphorylation changes within the immunity network. Analysis of this dataset provides key dynamic phosphorylation changes at early time points in the flg22 pathway while also providing a broader picture of overarching changes made in the root in response to flg22. In conjunction with cell biology and biochemical validation, this report reveals a phosphatase of AtRGS1 that plays an integral role in flg22-induced AtRGS1 phosphorylation, endocytosis, and degradation and, therefore, a key player in G signaling activation and dynamics.

## Experimental Procedures

### Plant Genotypes and Growth Conditions

Arabidopsis (*Arabidopsis thaliana*) ecotypes for all plants used in this study are Columbia-0 (Col-0). The *gpa1-4*, *agb1*-2, *agg1*-1, and *agg2*-1 quadruple mutant (hereafter *quad* mutant) was described in (30). The *rgs1-2* (SALK_074376.55.00) protein-null, T-DNA insertion mutant was created as previously described (6). The *fls2-1* (SAIL_691_C4) protein-null, T-DNA insertion mutant was created as previously described (31). The AtRGS1-YFP reporter was combined with *abi2-*1 (SALK_015166C), *abi2-*2 (SAIL_547_C10), *atbα-1* (SALK_032080C) (32), *atbα-2* (SALK_090040), *atbα-*3 (SALK_027428), *atbα-*5 (Wiscseq_DsLox340A05.0), *atbα-*6 *(*Wiscseq_DsLoxHs084_08E.1), *topp8-*2 (SALK_125184), *topp8-*4 (SALK_076144), *dsp1-*3 (WiscDsLo473B10), *dsp1-*4 (SAIL_116_C12), and *atbβ-*1 (GK-290G04-01) mutant backgrounds through crossing to create stable transformants. Genotyping utilized primers provided in supplemental Table S1 along with the T-DNA insertion positions for the phosphatase mutants used here.

Unless otherwise described, seeds were surface sterilized with 70% ethanol for 5 min while vortexing followed by a 5-min treatment with 95% ethanol. Seeds were subsequently washed 3X with ultrapure dH20 and suspended in 12-well cell culture plates with ¼ MS with no sugar at pH 5.7 or plated in similar media with 0.8% agar added. Plates were wrapped in aluminum foil, cold-treated at 4 °C for 2 days prior to germination.

For phosphoproteomics analysis, *A. thaliana* accession (Col-0) was used as WT while the quadruple (*quad*) mutant consisted of *gpa1-4*, *agb1-2*, *agg1-1*, and *agg2-1* alleles. After the surface-sterilized seeds were placed on sterile nylon mesh (Amazon, Nylon 6/6 Woven Mesh Sheet, Opaque Off-White, 40" Width, 10 yards length, 110 microns mesh size# B0013HNZJC), overlaying Murashige and Skoog basal agar media with 0.5% sucrose and stratified at 4° C for 2 days in the dark. Plants were then grown for 12 days in a growth chamber with 24-h constant light at the intensity of 150 photons per m^2^. After 12 days, 10 ml of the mock (water) or flg22 solution was added directly on the roots and kept submerged for 3 min and 15 min. Root tissue was then harvested and flash frozen in liquid nitrogen. Prior to protein extraction, the tissues were ground for 15 min under liquid nitrogen using a mortar and pestle. A comparison of the experimental design used here with similar studies is shown in supplemental Table S2.

### Chemicals

Methyl-β-cyclodextrin was purchased from Frontier Scientific, and tyrphostin A23 (TyrA23) was purchased from Santa Cruz Biotechnology. All chemicals were indicated by the vendors to be >98% pure.

### Quantification of AtRGS1 Abundance and Phosphorylation Levels *via* Immunoblot Analysis

In order to identify protein phosphorylation at the C-terminal serine cluster of AtRGS1, seven-day-old *rgs1*-2 seedlings overexpressing TAP-tagged AtRGS1 were treated with 100 nM flg22 for 0, 3, and 15 min. Total protein was extracted as described by (15). AtRGS1 was purified using IgG-agarose beads (Sigma), and the protein A portion of TAP tag was then partially removed by TEV digestion. Phosphorylation levels at the serine cluster were determined using an anti-phospho-AtRGS1 antibody, which recognizes the pSer428, pSer435, and/or pSer436 with low avidity (10). Total AtRGS1 levels were determined using an anti-AtRGS1 antibody (9272). Both sera were produced at the University of North Carolina and are available from AgriSera AB. Detection of the phosphorylated AtRGS1 protein *in vivo* in the atbα mutant or in *in vitro* in enriched samples was not possible due to the greatly reduced abundance of AtRGS1 protein.

For the stability assay, 7-day-old seedlings were treated with the translation inhibitor cycloheximide at 200 μM for 1 h. Protein levels were compared by Ponceau S staining and AtRGS1 levels were determined by probing with anti-GFP Tag polyclonal antibody (Invitrogen #A-11122). Bands were quantified using the software ImageJ (https://imagej.net/ij/) and the RuBisCO large chain (rbcL) was used as an endogenous control of total protein levels.

### Imaging ROS with Confocal Microscopy

Chloromethyl 2′,7′-dichlorodihydrofluorescein diacetate, H2DCF-DA (Thermo Fisher Scientific), was used as a generic reactive oxygen species (ROS) sensor (33). H2DCF-DA was prepared as previously described (34, 35). Briefly, H2DCF-DA dissolved in dimethyl sulfoxide to yield a 50 mM stock and diluted in deionized water to yield a final concentration of 6.25 μM. Roots were incubated with flg22 for the indicated time prior to a brief washout with water and a 10-min incubation with the H2DCF-DA solution.

2′,7′ -dichlorofluorescin (DCF) fluorescence was imaged on a Zeiss 880 laser scanning confocal microscope and excited with 0.2% maximum laser power at 488 nm with a 2.0 digital gain and a Plan-Neofluar 20×/0.50 Ph2 objective lens. The DCF signal was collected between 495 and 550 nm with a pinhole yielding 1 Airy Unit, making sure to limit excess exposure to the laser that induces ROS. Maximum intensity projections were produced from Z-stacks. All micrographs within each panel were acquired using identical offset, gain, and pinhole settings using the same detectors. DCF fluorescence intensities were measured in the maximum intensity projections using Fiji ImageJ by placing an ROI around the elongation zone near the root tip. The average intensity values within each ROI were recorded and averaged.

### Protein Extraction and Digestion

The proteomics experiments were carried out based on established methods (30, 36). Protein was extracted and digested into peptides with trypsin and Lys-C using the phenol-FASP method as previously detailed (30). Resulting peptides were desalted using 50 mg Sep-Pak C18 cartridges (Waters), dried using a vacuum centrifuge (Thermo Fisher Scientific), and resuspended in 0.1% formic acid. Peptide amount was quantified using the Pierce BCA Protein assay kit.

### Tandem Mass Tag Labeling

The tandem mass tag (TMT) labeling strategy used in this experiment is provided in supplemental Table S3. Approximately 40 μg of peptides were taken from each individual sample and then pooled. TMTpro 16plex labeling reagents (Thermo Fisher Scientific, Lot UH290430) were used to label 150 μg of peptides, from each sample or pooled reference at a TMT:peptide ratio of 0.2:1 as described in (37). After 2 h incubation at room temperature, the labeling reaction was quenched with hydroxylamine. Next, the 16 samples were mixed together and stored at −80 ºC until phosphopeptide enrichment. Labeling efficiency was checked by performing a 60-min 1D run on 200 ng of TMT-labeled peptides. All samples had labeling efficiencies ≥97%.

### Phosphopeptide Enrichment

The TMT-labeled phosphopeptides were first enriched using the High-Select TiO_2_ Phosphopeptide Enrichment Kit (Thermo Fisher Scientific) using the manufacturer’s protocol. The High-Select Fe-NTA Phosphopeptide Enrichment Kit (Thermo Fisher Scientific) was then used on the flowthrough from the TiO_2_ enrichment to enrich additional phosphopeptides. The manufacturer’s protocol for the Fe-NTA kit was used except the final eluate was resuspended with 50 μl 0.1% formic acid. The eluates from the TiO_2_ and Fe-NTA enrichments were combined and stored at −80 ºC until analysis by LC-MS/MS.

### LC-MS/MS

An Agilent 1260 quaternary HPLC was used to deliver a flow rate of ∼600 nl min-1 *via* a splitter. All columns were packed in house using a Next Advance pressure cell, and the nanospray tips were fabricated using a fused silica capillary that was pulled to a sharp tip using a laser puller (Sutter P-2000). Ten micrograms of TMT-labeled peptides (non-modified proteome), or ∼fifteen micrograms of TiO_2_ or Fe-NTA enriched peptides (phosphoproteome), were loaded onto 10-cm capillary columns packed with 5 μM Zorbax SB-C18 (Agilent), which was connected using a zero dead volume 1 μm filter (Upchurch, M548) to a 5-cm long strong cation exchange (SCX) column packed with 5 μm polysulfoethyl (PolyLC). The SCX column was then connected to a 20-cm nanospray tip packed with 2.5 μm C18 (Waters). The three sections were joined and mounted on a Nanospray Flex ion source (Thermo Fisher Scientific) for on-line nested peptide elution. A new set of columns was used for every sample. Peptides were eluted from the loading column onto the SCX column using a 0 to 80% acetonitrile gradient over 60 min. Peptides were then fractionated from the SCX column using a series of 17 and 8 salt steps (ammonium acetate) for the nonmodified proteome and phosphoproteome analysis, respectively. For these analyses, buffers A (99.9% H_2_O, 0.1% formic acid), B (99.9% ACN, 0.1% formic acid), C (100 mM ammonium acetate, 2% formic acid), and D (2 M ammonium acetate, 2% formic acid) were utilized. For each salt step, a 150-min gradient program comprised of a 0 to 5 min increase to the specified ammonium acetate concentration, 5 to 10 min hold, 10 to 14 min at 100% buffer A, 15 to 100 min at 15 to 30% buffer B, 100 to 121 min at 30 to 45% buffer B, 120 to 140 min at 45 to 80% buffer B, 140 to 144 min at 80% buffer B, and 145 to 150 min at buffer A was employed.

Eluted peptides were analyzed using a Thermo Scientific Q-Exactive Plus high-resolution quadrupole Orbitrap mass spectrometer, which was directly coupled to the HPLC. Data-dependent acquisition was obtained using Xcalibur 4.0 software (https://www.thermofisher.com/order/catalog/product/OPTON-30965) in positive ion mode with a spray voltage of 2.20 kV and a capillary temperature of 275 °C and an RF of 60. MS1 spectra were measured at a resolution of 70,000, an automatic gain control of 3e6 with a maximum ion time of 100 ms, and a mass range of 400 to 2000 m/z. Up to 15 MS2 were triggered at a resolution of 35,000 with a fixed first mass of 120 m/z for phosphoproteome and 120 m/z for proteome. An automatic gain control of 1e5 with a maximum ion time of 50 ms, an isolation window of 1.3 m/z, and a normalized collision energy of 31. Charge exclusion was set to unassigned, 1, 5–8, and >8. MS1 that triggered MS2 scans were dynamically excluded for 45 or 25 s for phospho- and non-modified proteomes, respectively.

### Phosphoproteomics Data Analysis

The raw spectra were analyzed using MaxQuant version 1.6.14.0 (38). Spectra were searched using the Andromeda search engine in MaxQuant (39) against the Tair10 proteome file containing 35,386 proteins entitled “TAIR10_pep_20101214” that was downloaded from the TAIR website (https://www.arabidopsis.org/download_files/Proteins/TAIR10_protein_lists/TAIR10_pep_20101214) and was complemented with reverse decoy sequences and common contaminants by MaxQuant. Carbamidomethyl cysteine was set as a fixed modification while methionine oxidation and protein N-terminal acetylation were set as variable modifications. For the phosphoproteome, “Phospho STY” was also set as a variable modification. The sample type was set to “Reporter Ion MS2” with “16plex TMT selected for both lysine and N-termini.” Digestion parameters were set to “specific” and “Trypsin/P;LysC.” Up to two missed cleavages were allowed. A false discovery rate, calculated in MaxQuant using a target-decoy strategy (40) at which a value of less than 0.01 at both the peptide spectral match and protein identification level was required. The ‘second peptide’ option identify cofragmented peptides was not used. The match between runs feature of MaxQuant was not utilized. In MaxQuant, we specified that the only nonphosphopeptides were used for protein quantification and that the unmodified form of a phosphopeptide was discarded. Phosphorylation sites were localized to a given amino acid using the MaxQuant localization probability score.

Statistical analysis on the MaxQuant output was performed using the TMT-NEAT Analysis Pipeline (https://github.com/nmclark2/TMT-NEAT). TMT-NEAT was used for sample loading (within-run) and internal reference (between-run) normalization to eliminate batch effects (41) to generate quality control plots such as *p*- and *q*-value histograms and to determine differential expression with PoissonSeq (42). Protein groups and phosphosites were categorized as differentially accumulating in each pairwise comparison to a matched control if they were under a false discovery rate cutoff of *q-*value <0.05.

### Phosphosite Overlap with the G-Signaling Interactome

The locus codes for those genes were then compared to the Arabidopsis G-signaling interactome database (AGIdb, http://bioinfolab.unl.edu/AGIdb) and additional interactions were identified by the Arabidopsis Interactions Viewer (http://bar.utoronto.ca/interactions2/). A network was created using the software Cytoscape (https://cytoscape.org/).

### Gene Ontology Analysis

The lists generated by phosphoproteomic profiling were combined into two lists: (1) proteins containing increased or (2) decreased phosphosite abundance in Col-0. The two lists were submitted to PANTHER Go-Slim molecular function analysis for an overrepresentation test and fold-enrichment values were determined in comparison with the complete Arabidopsis Gene Database. Significant GO terms had a corrected *p-*value <0.05.

### Interactome Construction and Analysis

The Arabidopsis experimental protein-protein interaction (PPIE) network was curated from STRING (with experimental evidence) (43), Arabidopsis interactome map (AI-1MAIN) (44), plant-pathogen immune network (PPIN-1 and 2) (29, 45), cell surface interactome (46), literature-curated interactions (47), membrane-linked Interactome Database version 1 (MIND1) (48), EffectorK (49), and BioGRID (50). The collective PPIE was used to extract the significantly phosphorylated proteins (q_value <0.05) interactors using in-house python scripts. The network was imported to Cytoscape (ver. 3.9.0.) for topology centrality analysis and visualization (51). To identify the highly connected phosphorylated proteins in the PPIE network, we selected the degree centrality cutoff of ∼top 5% of nodes (degree ≥15; hub^15^) as described in (26, 29). The functional enrichment analysis was performed by Metascape (52). The locus codes for those genes were then compared to the Arabidopsis G-signaling interactome database (AGIdb, http://bioinfolab.unl.edu/AGIdb) and additional interactions were identified by the Arabidopsis Interactions Viewer (http://bar.utoronto.ca/interactions2/). The network was created using the software Cytoscape.

### Quantifying AtRGS1-YFP Internalization

AtRGS1-YFP internalization was induced with flg22 as described (10, 13, 18). Briefly, 3-5-day-old, etiolated Arabidopsis seeds expressing 35S:AtRGS1-YFP were treated with flg22 for 2, 10, or 30 min respectively before imaging. Image acquisition was done on a Zeiss LSM880 (Zeiss Microscopy) with a C-Apochromat 40×/1.2NA water immersion objective. YFP excitation was at 514 nm and emission collection was at 525 to 565 nm. Emission collection was performed with a GaAsP detector. Z-stack series were acquired at 0.5 μm intervals between images with a pinhole yielding 1 Airy Unit. Image processing and RGS internalization measurements were performed with Fiji ImageJ. Internalized YFP fluorescence was measured and subtracted from total YFP fluorescence of individual cells. Images were acquired on the hypocotyl epidermis at 2 to 4 mm below the hypocotyls. Seedling exposure to light was minimized as much as was practical while imaging.

### Pharmacological Inhibition of Baseline AtRGS1-YFP Internalization and Protein Production in Atbα

Etiolated seedlings (3–5 day-old) were preincubated with 50 μM TyrA23 and methyl-β-cyclodextrin (MβCD) 5 mM for 2 h as previously described (18). Seedlings were briefly washed with water and transferred to liquid growth media for 0, 2, 10, 30, or 60 min prior to imaging. For coincubation with endocytosis inhibitors and cycloheximide, seedlings were incubated with TyrA23 and MβCD for 2 hours prior to incubation with TyrA23, MβCD, and 200 μM cycloheximide for 1 h. Imaging AtRGS1-YFP was performed as described above.

### Firefly Split Luciferase Assay

pCAMBIA/des/cLuc and pCAMBIA/des/nLuc were used to generate the following plasmids: ATBα-nLUC and ATBβ-nLUC. AtRGS1-nLUC, cLUC-AtGPA1, and AGB1-nLUC were previously reported (18). Expression was normalized by HiBiT (53). pART27H-mCherry-AtAGG1 plasmid was obtained from Dr Jose R Botella (University of Queensland). All plasmids were transformed into *Agrobacterium tumefaciens* strain *GV3101*. nLUC and cLUC fusion partners were co-expressed in *Nicotiana benthamiana* leaves by agroinfiltration following protocols (54). Forty-eight hours after infiltration, 6-mm leaf discs were collected to 96-well plate, abaxial side down, and 40 μl 0.4 mM D-luciferin was added to each well. Luminescence was measured by a spectraMax L microplate reader (Molecular Devices).

### Kinase Inhibition Assay

GST-WNK8 (0.5 ug), of GST-ATBα (5 μg) and/or 10 μg of 6XHis-RGS1 C terminal half (J5), as described by (10) were incubated in 15 μl of kinase reaction buffer (5 mM Tris–HCl pH 7.5, 1 mM MgCl2, 0.4 mM ATP, 1 mM PMSF) with a radio-labeled [γ-32P]-ATP at 20 °C for 4 h. The samples were separated on a SDS-PAGE gel and exposed on an intensifying screen. The relative amounts of ^32^P were quantitated and provided as relative values. To test for an effect of the GST tag on RGS1 J5 phosphorylation, GST-RACK1 was included and shown to have no effect.

### Luminol-Based ROS Analysis

flg22-induced ROS bursts were measured as described (9, 55). Briefly, leaf discs from 5-week-old plants were placed singly abaxial-side down into a 96-well plate with 250 μl of water per well. After overnight incubation, the water was replaced with 100 μl of reaction mix (17 μg/ml of Luminol (Sigma), 10 μg/ml of horseradish peroxidase (Sigma), and 100 nM flg22). Luminescence was measured immediately with 1 s integration and 2 min intervals using a SpectraMax L (Molecular Device).

### Hypocotyl Elongation Assay

The number of hypocotyl epidermal cells in a flank are determined during embryogenesis (56). Etiolated hypocotyl length up to approximately 2 days after germination is determined, in part, by this cell number in the G protein mutants (57). Thus, hypocotyl length under these conditions is a proxy for embryonic cell division and should not be confused with assays measuring hypocotyl length of >5-day-old seedlings where intercalary meristematic activity becomes important in the final organ length (*e.g.* 58).

The hypocotyl assay was performed as previously described (57, 59) with some exceptions. Briefly, matched seeds of Col-0 and null mutants were sterilized, then germinated on square plates with ½ x MS medium, pH 5.7, 0.8% (w/v) agar, supplemented with 1% (w/v) sucrose, and stratified for 4 days. Plates were light treated for 4 hours to induce and synchronize germination, wrapped in aluminum foil, and covered to grow in the dark for 64 h at 25 °C. Hypocotyls were imaged with a Nikon digital camera (D40) against a black background and quantified with Fiji ImageJ. Germination rates were performed as described by (60) and are provided in Supplemental Information.

### Real Time RT-qPCR

RNA was extracted from seedlings grown on ¼ x MS medium, pH 5.7, 0.8% (w/v) agar, and 1% (w/v) sucrose for 1 week. The complimentary DNA library was prepared as a 1:1 mixture of oligo(dT) and random hexamer primers and maxima reverse transcriptase enzyme (Thermo Fisher Scientific, Reference: EP0742). RT-qPCR analysis using this complimentary DNA was performed on a MJ Research DNA Engine Opticon 2: Continuous Fluorescence Detector, using SYBR Green detection chemistry. Primers from Eton Bioscience Inc were used to amplify a 200 to 300 bp amplicon 3′ of all T-DNA insertions (supplemental Table S1). TUBULIN4 primers [Fwd: AGAGGTTGACGAGCAGAT; Rev: ACCAATGAAAGTAGACGC] were used as an internal control to account for amount or RNA extracted across mutants. C(t) values were used to calculate fold-induction results, which were normalized to Col-0.

### Experimental Design and Statistical Rationale

**Fig. 1:**
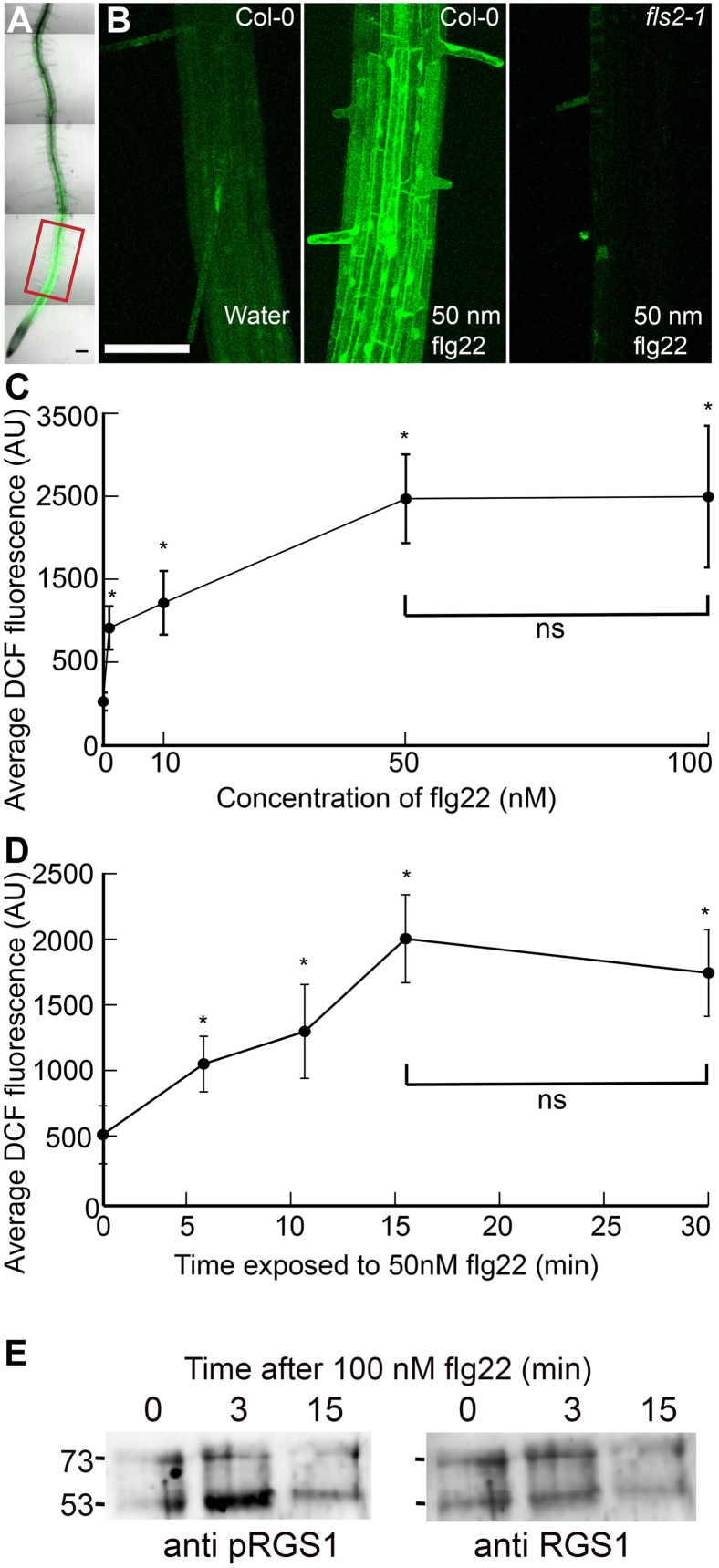
**Dose and time effects of flg22 on ROS bursts in primary roots.***A*, confocal micrograph showing flg22-induced ROS bursts by DCF staining of *Arabidopsis* primary root. *Red* box indicates region of interest used to quantify fluorescence. *B*, confocal micrographs of the elongation zone of primary roots stained with DCF ± flg22. *C*, quantification of DCF-stained root tips showing dose response to different concentrations of flg22. *D*, quantification of DCF-stained root tips showing timing of flg22-induced ROS bursts. Error bars represents SD. *E*, flg22-induced phosphorylation of AtRGS1. Ten-day-old *rgs1-2* mutant seedlings expressing AtRGS1-TAP were treated with 100 nM flg22 for 0, 3, and 15 min. AtRGS1 was purified using IgG-agarose chromatography and the TAP tag partially removed by TEV protease digestion and subsequently detected by Western blot analysis using an anti-phospho-AtRGS1 antibody (*left* blot) and reprobed with anti-AtRGS1 antibody (*right* blot) for loading. Molecular weight of 53 and 73 kDa are shown at the *left*. TEV-cleaved AtRGS1 of the correct size is the *lower* band in the doublet and the uncleaved AtRGS1-TAP protein is the *upper* band. *Asterisks* in *panels C* and *D* indicate significant difference (*p* < 0.01) between water and flg22 treatment determined by a two-way ANOVA followed by Tukey’s post hoc test. Scale bars represent 100 μm. DCF, 2′,7′ –dichlorofluorescin.

In Figures 1, 3, 4, and 5*A*, one-way ANOVA with post hoc Tukey HSD analysis was performed using GraphPad Prism 7. In Figure 4*F*, statistical differences between groups were established using unpaired *t* test and in Figure 5*B*, using a one-way ANOVA with a post hoc test using a Bonferroni approach. The gene ontology overrepresentation test was Fisher's Exact with Bonferroni correction for multiple testing. Three independent biological replicates for each genotype and treatment, described above (plant genotypes and growth conditions) were profiled for the phosphoproteome analyses. Statistical analysis of these samples is described above in the Phosphoproteomics Data Analysis subsection.

## Results

### Dose Response and Time Dynamics of flg22-Induced ROS Bursts in Roots

It is well established that root tissue responds to flg22. Emonet and co-workers (61) showed that FLS2 is expressed in the epidermis and cortical cells throughout the root except the tip. Their paper also showed that many cell types can respond, some autonomously, to flg22. Our previous baseline analysis (no flg22) (30) utilized roots. Moreover, sampling roots grown as described in the Experimental Procedures is more suitable for the time resolution required here because treated roots can be collected quickly. G proteins mediate numerous root responses and are well established for root tissue (15, 62, 63, 64, 65, 66). These are the reasons that root tissue was chosen for this study.

Our strategy was to use quantitative proteomics in root cells to elucidate changes in the phosphorylation state caused by G protein–dependent flg22 signaling. This first required establishing the kinetics of flg22 signaling in WT roots to determine the most informative dose and time for the flg22 response profiled in subsequent experiments. Thus, we employed confocal microscopy to visualize peak flg22 signaling in roots using ROS bursts as a marker for flg22 signaling. To visualize ROS, Arabidopsis roots were treated with the ROS sensor H2DCF-DA. The detected intracellular ROS distribution was highest in the elongation zone (Fig. 1*A*, red box). DCF fluorescence was enhanced in most cells of Col-0 roots treated with 50 nM flg22 compared to those treated with water (Fig. 1*B*, cf. left panel to middle panel) indicating that the root is highly responsive to flg22. In contrast to WT roots, no increase in fluorescence was observed in flg22-treated *fls2-1*, a null mutant of the flg22 receptor, FLS2 (Fig. 1*B*, cf. middle panel to right panel) indicating that flg22-induced ROS production is mediated entirely by the FLS2 pathway. The flg22-induced ROS burst dose response reached saturation between 10 and 50 nM (Fig. 1*C*). To determine the optimal treatment time for our phosphoproteomics experiment, we treated roots with 50 nM flg22 for 0, 5, 10, 15, and 30 min. Peak DCF fluorescence was observed at 15 min (Fig. 1*D*). Finally, we mapped the time course of phosphorylation of AtRGS1 (17) after flg22 treatment (100 nM). A clear increase in phosphorylation per protein at the previously-identified phosphocluster (10) was observed within 3 min and may have decreased by 15 min (Fig. 1*E*).

### Analysis of flg22-Induced G Protein–specific Phosphoproteome

**Fig. 2:**
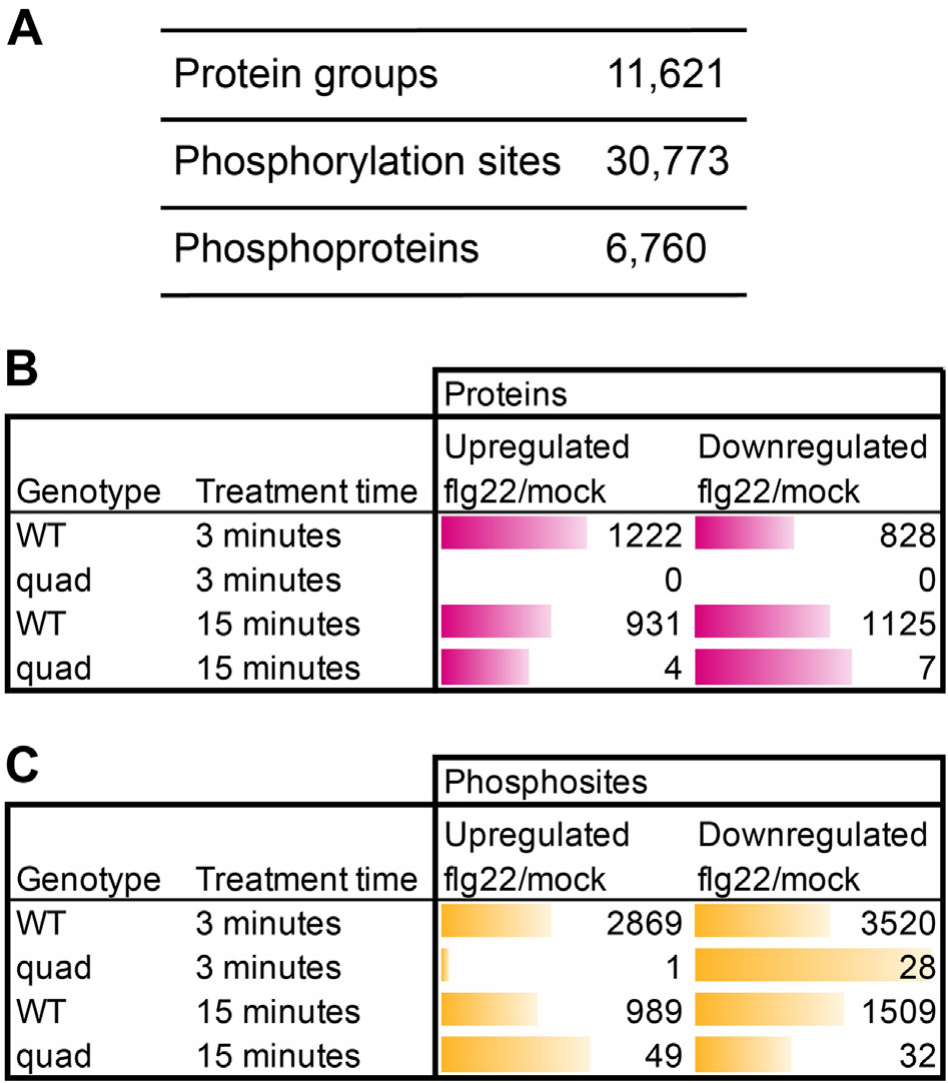
**Identification of flg22 responsive proteins and phosphorylation sites.***A*, the number of protein groups, phosphorylation sites, and phosphoproteins detected. *B*, number of differentially abundant (q < 0.05) phosphorylation sites in WT and *quad* mutant backgrounds after 3 or 15 min of flg22 treatment. *C*, number of differentially abundant (q < 0.05) proteins sites in WT and *quad* mutant backgrounds after 3 or 15 min of flg22 treatment.

Having established the time course and dose response of flg22-induced ROS production in roots, we treated 12-day-old WT Columbia (Col-0) as well as mutant plants deficient in Gα, Gβ, and two out of the three Gγ subunits in Arabidopsis (30): *gpa1-4*, *agb1*-2, *agg1*-1, and *agg2*-1 quadruple mutant (designated *quad* or G protein mutant hereafter) seedlings with water (mock) or 50 nM flg22 and collected samples at 0, 3, and 15 min to ensure captured of changes in the phosphoproteome as the flg22 response reaches its temporal peak. Protein abundance and phosphorylation level was quantified by performing two-dimensional liquid chromatography-tandem mass spectrometry on TMT (TMTpro) labeled peptides (30, 36, 67, 68). From these samples, we identified 11,621 proteins (8090 at 3 min and 9647 at 15 min) and 30,773 phosphorylation sites (24,575 at 3 min and 22,903 at 15 min) arising from 6760 phosphoproteins (Fig 2*A* and supplemental Datasets S1 and S2, and supplemental Fig. S1).

TMT-NEAT was used for statistical analyses (36) and assigned gene products as differentially expressed if they were under a false discovery rate cutoff of *q-*value <0.05 providing a stringent method for discovering statistical differences (see Experimental Procedures section). Because different subsets of proteins and phosphorylation sites were detected at each time point, we focused the following comparative analyses within each time point. In WT plants, 6389 sites corresponding to 2197 phosphoproteins and 2498 sites corresponding to 1062 phosphoproteins were differentially phosphorylated following 3 min and 15 min, respectively, of flg22 treatment compared to the paired mock (H_2_O) samples. In the absence of the heterotrimeric G protein components (in the *quad* mutant), only 29 and 81 sites (corresponding to 14 and 36 phosphoproteins) are differentially phosphorylated after 3 and 15 min of treatment, respectively, suggesting that the majority of the flg22-regulated sites are G-protein–dependent (Fig 2*C*, supplemental Dataset S1, and supplemental Fig. S1*B* Volcano Plots). The results for protein abundance were similar, with a large number of protein groups differentially expressed after 3 min and 15 min of flg22 treatment in Col-0 (2050 and 2,056, respectively) but minimal changes in protein abundance in the *quad* mutant (0 and 11, respectively) (Fig. 2*B* and supplemental Dataset S2). These results also indicate that the vast majority of flg22-induced proteome remodeling requires a functional G protein complex.

### Pathogenic Effectors Preferentially Target Phosphoproteins

**Fig. 3:**
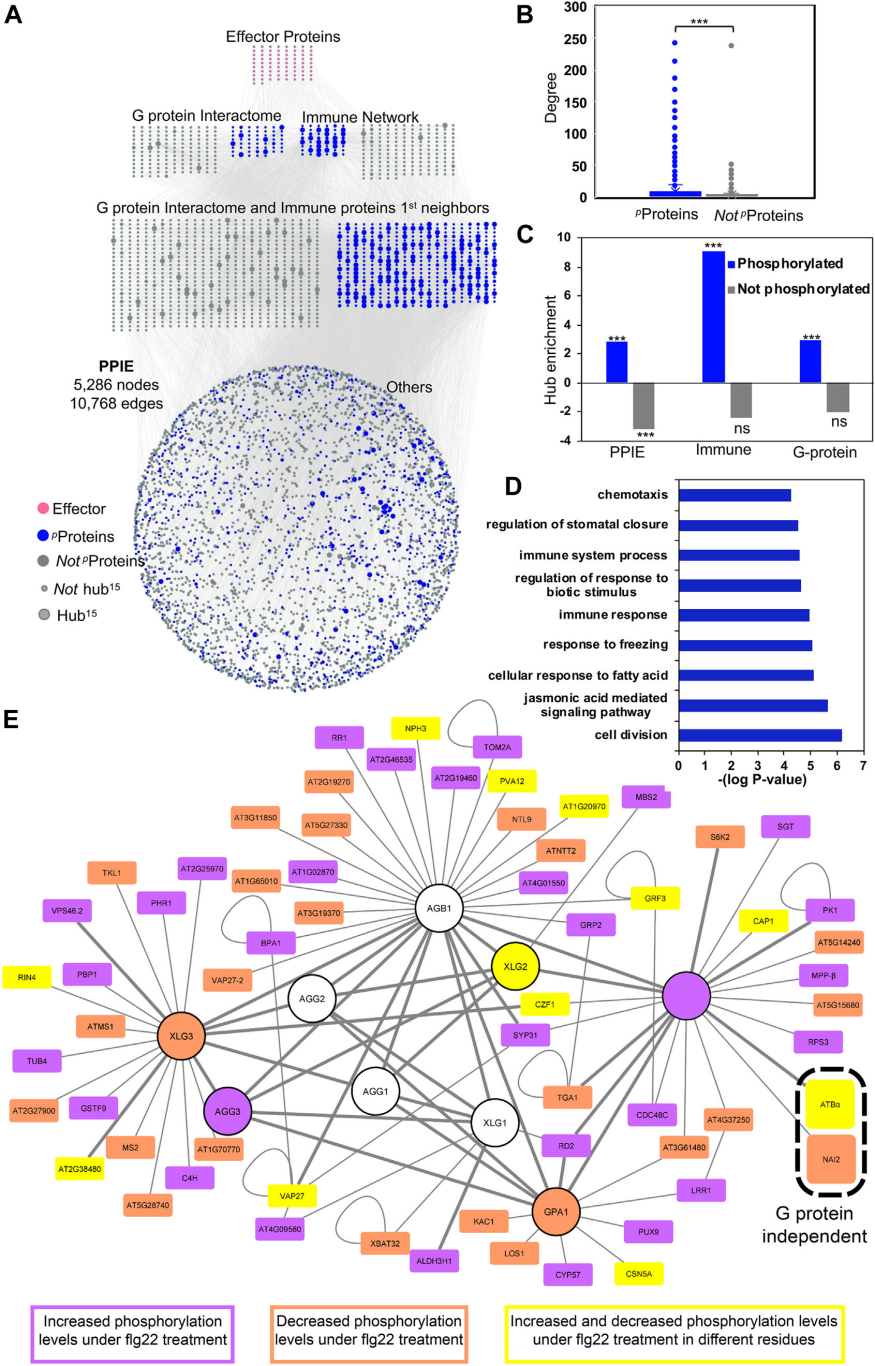
**Pathogen-induced phosphoproteins are preferentially selected by effector proteins.***A*, the Arabidopsis experimental PPI (PPIE) network with 5286 nodes and 10,768 interactions. (*Pink* = effectors, *blue* = phosphorylated proteins (^*p*^Proteins), *gray* = not phosphorylated proteins (*Not*^*p*^Proteins), large size= hub^15^, small= not hub^15^). *B*, the degree distribution of phosphorylated illustrates significantly high degree nodes than that of nonphosphorylated proteins in PPIE (Student’s *t* test *p*-value ≤0.001). *C*, the significant hub enrichment of phosphorylated proteins and not phosphorylated proteins in PPIE, immune, and G protein interactome nodes (∗∗∗ hypergeometric test enrichment *p*-value ≤0.001, ns = not significant). *D*, GO-ontology term enrichment of immune and G protein interactome hub^15^ nodes (*p*-value ≤ 0.05). *E*, proteins from the Arabidopsis G Protein Interaction Database that respond to flg22 treatment. (mock vs. 3 or 15 min flg22, q < 0.1), having increased phosphorylation (*purple*), decreased phosphorylation (*orange*), or both (*yellow*) in different residues. The two highlighted proteins represent the only proteins that respond to flg22 treatment on the quad mutant. Thicker edges represent a higher confidence of interaction based on previously published physical interactions. Arabidopsis G protein interactome can be accessed at http://bioinfolab.unl.edu/emlab/Gsignal.

Highly connected nodes or hubs are preferential targets of pathogen effectors (29, 44, 45, 49); thus we performed network topology analysis of the significantly flg22-induced phosphoproteome (q_value <0.05) in the context of Arabidopsis PPIs. As described in the Experimental Procedures section, we used an expanded version of the experimental interactome (43) and integrated the 2620 flg22-induced phosphoproteins. The resulted network encompassed 5286 nodes with 10,768 interactions including interactions from the Arabidopsis immune network and G-protein interactome (Fig. 3*A* and supplemental Dataset S3 PPIE). Network centrality analysis revealed that phosphorylated proteins (^*p*^Proteins) have more interacting partners than nonphosphorylated proteins (*Not* ^*p*^Proteins) during the flg22-induced defense response (Fig. 3*B*, Student’s *t* test *p*-value ≤0.001). This analysis inferred that the *in* *planta* immune network and G-protein interactome utilize phosphorylation for efficacious downstream signaling during pathogen infection to modulate the host defense responses. Next, we computed the hubs (proteins with degree ≥15, designated “hub^15^”) in our flg22-induced PPIE. Hubs are significantly enriched among phosphorylated nodes (Fig. 3*C* and supplemental Dataset S3 PPIE, hypergeometric test *p*-value ≤0.001) i.e., the frequency of hub^15^ in phosphorylated nodes is statistically more prevalent than in nonphosphorylated nodes. Furthermore, we report that hub^15^ nodes are enriched in the immune network as a whole, which establishes our previous findings (26). Concomitantly, we also observed that hub^15^s are statistically more prevalent in phosphorylated nodes of the G-interactome and immune network, whereas they are under-enriched among the nonphosphorylated proteins during flg22 treatment (Fig. 3*C* and supplemental Dataset S3 PPIE, hypergeometric test *p*-value ≤0.001). Additionally, the GO-ontology terms of phosphorylated hubs in immune and G-protein interactome nodes revealed that most of these proteins are significantly involved in chemotaxis, stomata closure, immune systems process, regulation of biotic stimulus response, immune response, response to freezing, cellular response to fatty acids, jasmonic acid signal pathway, and cell division to name a few (Fig. 3*D* and supplemental Dataset S3 PPIE, *p*-value ≤ 0.05).

### Arabidopsis G Protein Interactome Contains Proteins that Respond to flg22

Figure 3*E* shows proteins in the Arabidopsis G-protein interactome that have flg22-induced changes in phosphorylation. Only two of these phosphoproteins show the same phosphorylation patterns on the *quad* mutant as in WT and both of them are AtRGS1 interaction partners (Fig. 3*E*, dashed oval outline) (19). The protein phosphatase 2A (PP2A) regulatory B subunit (ATBα) is one of the two proteins that have decreased phosphorylation levels under stress in a G protein–independent manner. PP2A is a widely conserved serine/threonine phosphatase found in both the heterodimer and heterotrimer forms (69). The PP2A heterotrimer is composed of the structural A subunit, the substrate-recognition B subunit (aka regulatory subunit), and the catalytic C subunit. ATBα, the alpha isoform of the B subunit, along with highly similar ATBβ make up a subfamily of B subunits that are dissimilar to all other isoforms of the B subunit (70). This finding raises the possibility that ATBα is one of the phosphatases in the G protein interactome that act antagonistically to the kinases that phosphorylate AtRGS1 in the presence of a signal (17).

Regarding the G protein complex, AtRGS1 phosphorylation at Ser430 decreased in the *quad* mutant 15 min post flg22 treatment; this site was not detected at 3 min (supplemental Dataset S2). The alpha subunit AtGPA1 showed decreased levels of phosphorylation on a plant-unique serine residue (Ser202) and the Gγ subunit AGG3 showed increased phosphorylation at Ser37 after 3 min of treatment. flg22-induced posttranslational modifications on G-proteins were found on extra-large G proteins (XLGs), where XLG2 showed a decreased phosphorylation level (q < 0.1) on several residues outside the Gα domain (Ser75, Ser184, Ser185, Ser190, Ser191, Ser194, and Ser198) and an increase phosphorylation on residues Ser13 and Ser38 after 3 min. pSer13 and pSer38 are novel flg22-induced phosphorylation sites in roots, while Ser141, Ser148, Ser150, and Ser151 were previously detected as BIK1 phosphorylation sites under flg22 treatment in Arabidopsis protoplasts (71). This signaling dependency of the canonical G proteins, together with the XLG major modifications, prompts the hypothesis of regulation by competition between the XLGs and AtGPA1 for the AGB1/AGG dimers (72).

### Biochemical Interaction Between ATBα Phosphatase and AtRGS1

**Fig. 4:**
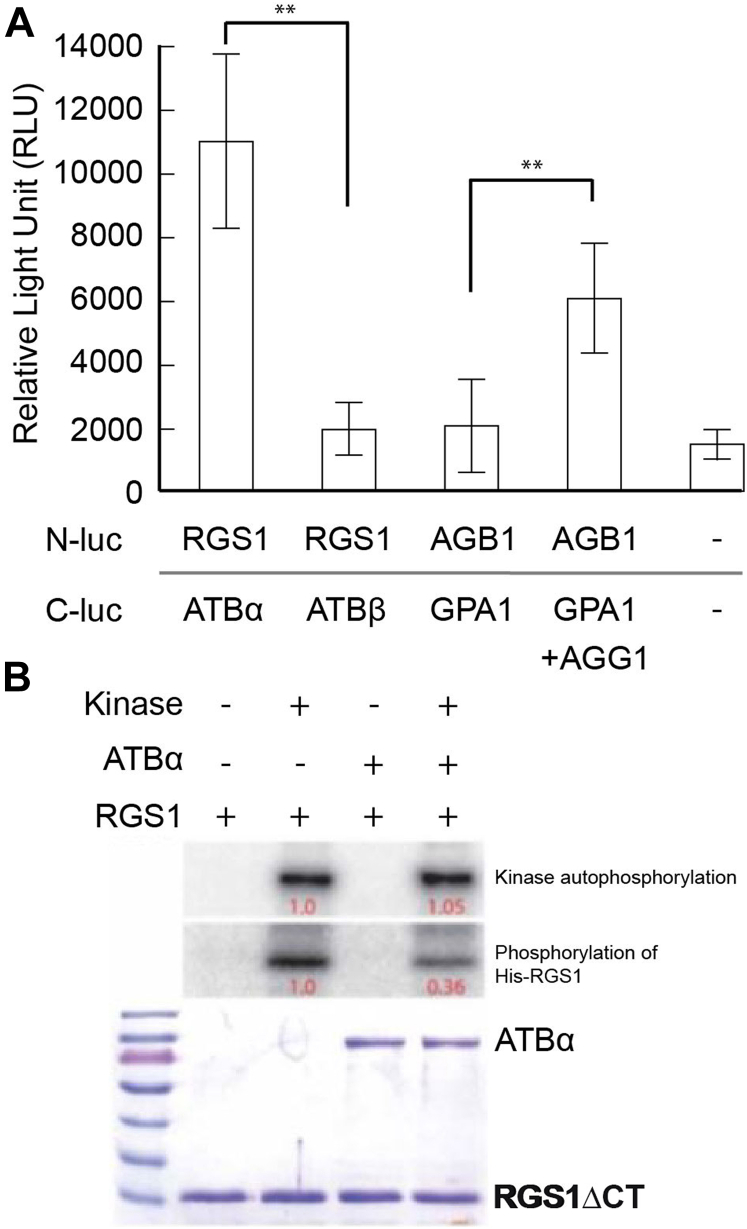
**ATBα but not ATBβ interact withAtRGS1.***A*, split luciferase assay showing protein interactions *in vivo*. ATBα but not ATBβ interacts with AtRGS1 (indicated RGS1). Positive control is complementation by the heterotrimeric G protein complex (AtGPA1/AGB1/AGG1). Negative control is AtGPA1 (indicated GPA1) and AGB1 in the absence of AGG1 as well as empty C- and N-luc vectors. Expression was normalized by HiBit. Graphs are representatives of three experimental replicates. Error bars represent confidence intervals (CI). Asterisks indicate significant difference (∗∗ *p* < 0.01) determined by a two-way ANOVA followed by Tukey’s post hoc test. n = 36. *B*, ATBα decreases AtRGS1 phosphorylation catalyzed by a WNK-family kinase. 0.5 μg of GST-WNK8, 5 μg of GST-ATBα, and/or 10 μg of His-RGS1 were incubated in a 15 μl of kinase reaction buffer (5 mM Tris–HCl pH 7.5, 1 mM MgCl2, 0.4 mM ATP, 1 mM PMSF) with a radio-labeled [γ-^32^P]-ATP at 20 °C for 4 h. The samples were separated on a SDS-PAGE gel and exposed on a PHOSPHOR screen. The PHOSPHOR screen image and the Coomassie-stained gel are shown. The relative amounts of ^32^P were quantitated and provided as relative values in this figure (*red*). AtRGS1 (RGS1) is poly His tagged and ATBα is GST tagged. The GST controls are shown in Supplemental Information as supplemental Fig. S3. WNK, with no lysine.

Because there is no information about AtRGS1 phosphatases in the AtRGS1 phosphorylation-dephosphorylation cycle, we chose to examine more closely ATBα, a substrate recognition subunit of PP2A. Its physical interaction with AtRGS1 *in planta* was tested with the Firefly Split Luciferase assay. As a negative control, we tested the interaction of AtGPA1 with AGB1 lacking its obligate Gγ subunit and observe no interaction (Fig. 4*A*, equaled basal level). As a positive control, we used the interaction of the G protein heterotrimer subunits. The GST control is shown in supplemental Fig. S2. AtRGS1 interacted with ATBα but not the ATBβ isoform, suggesting that the ATBα interaction with AtRGS1 is isoform-specific. To confirm this physical interaction and address specificity, we tested biochemical interaction *in vitro*. We chose the well-characterized phosphorylation reaction by its kinase WNK8 (10). In the absence of ATBα, WNK8 bound to and phosphorylated AtRGS1 as previously reported. However, the addition of the ATBα substrate-recognition subunit of this phosphatase blocked phosphorylation of AtRGS1 as expected for a substrate recognition subunit (Fig. 4*B*). One interpretation of this observation is that ATBα is binding to its substrate and blocking phosphorylation. In this interpretation, we posit that ATBα is a substrate-recognition subunit involved in the AtRGS1 phosphorylation-dephosphorylation cycle of the Ser/Thr phosphocluster necessary for endocytosis. The reciprocal experiment showing that the PP2A–ATBα phosphatase complex dephosphorylates AtRGS1 is hampered by the heterotrimeric property of PP2A making it difficult to reconstitute a functional phosphatase *in vitro* (70).

**Fig. 5:**
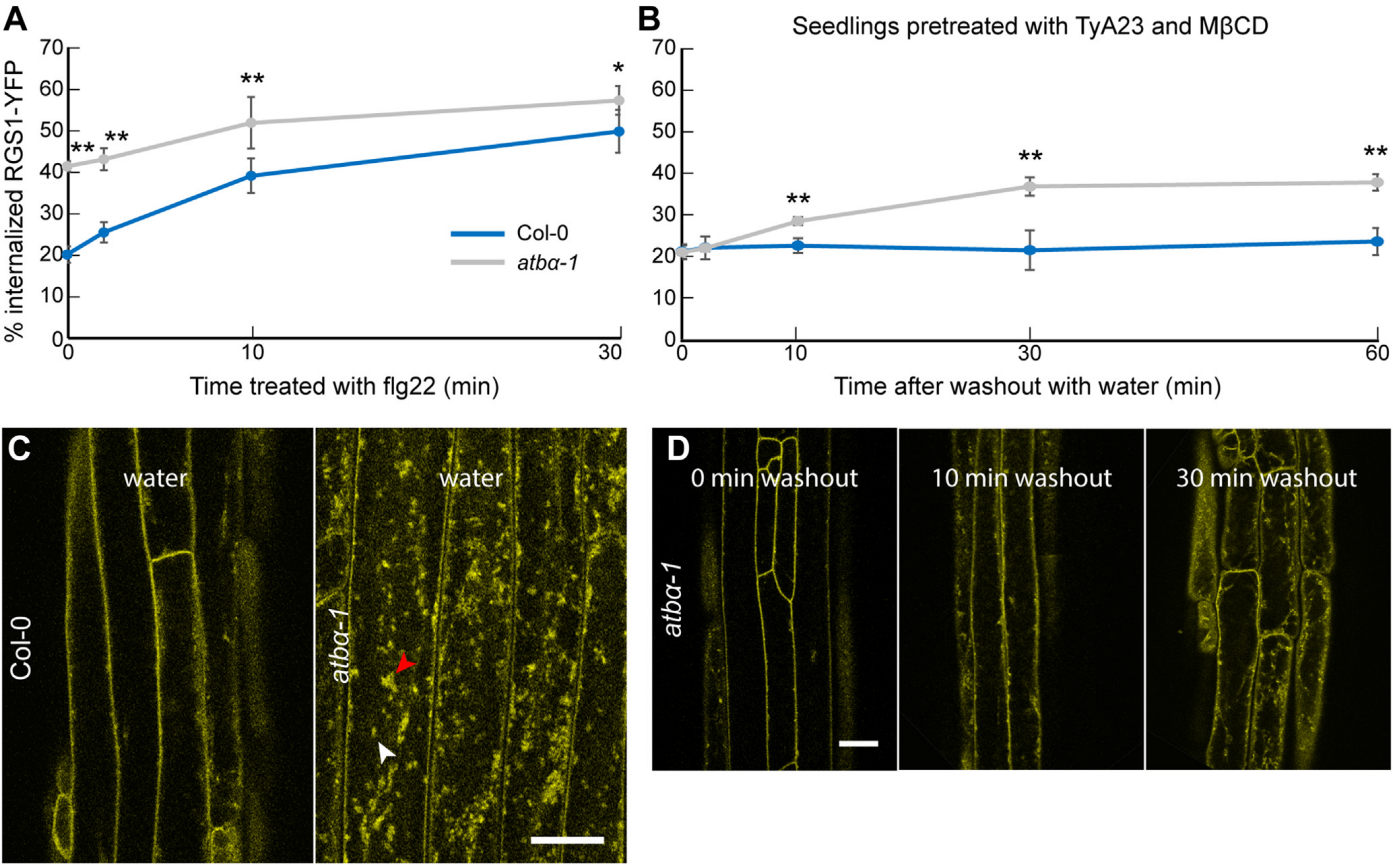
**ATBα modulatesAtRGS1 internalization in etiolated hypocotyl cells.***A*, flg22-induced AtRGS1-YFP internalization measured over time in Col-0 and *atbα-1* null mutant. ∗∗ and ∗ represents statistical significance (*p* < 0.01 or *p* < 0.05 respectively) between Col-0 and *atbα-1* at the indicated time point. Error bars represent SEM. n = 30 across three separate experimental replicates. *B*, Percent internalized AtRGS1-YFP measured in Col-0 and *atbα-1* measured over time after washout of internalization inhibitors with water. Col-0 seedlings were incubated in water for 2 h and *atbα-1* seedlings were incubated for 2 h with 50 μM TyrA23 (endocytosis inhibitor) and 5 mM MβCD (lipid raft inhibitor). ∗∗ and ∗ represents statistical significance (*p* < 0.01 or *p* < 0.05 respectively) between Col-0 and *atbα-1* at the indicated time point. Error bars represent SEM. n = 32 to 48 across three separate experimental replicates. The two pools of AtRGS1, one inhibited by TyrA23 and the other by MβCD, in flg22 are fully described in Watkins, *et al* (18). *C*, representative confocal micrographs of *atbα-1* quantified in panel *B*. Scale bar represents 20 μm. *D*, confocal micrographs of *atbα-1* expressing RGS1-YFP. Scale bar represents 20 μm. MβCD, methyl-β-cyclodextrin.

To summarize, four independent results indicate interaction between AtRGS1 and ATBα: (1): ATBα and AtRGS1 directly interact physically in Y2H (19); (2): AtRGS1 and ATBα interact physically (directly or indirectly) *in vivo* with SFLA in a specific manner, i.e., no interaction observed with the ATB isoform ATBβ (Fig. 4*A*); (3): AtRGS1 and ATBα physically interact specifically through blocking RGS1 phosphorylation by WNK8 kinase and this interaction is isoform-specific (Fig. 4B); and as will be addressed below, 4: AtRGS1 and ATBα interact genetically (Figs. 5 and 7).

### Genetic Interaction Between AtRGS1 and the ATBα Phosphatase

Having shown *in vivo* and *in vitro* biochemical interaction between ATBα and AtRGS1, we tested genetic interaction. A quantifiable property of AtRGS1 is internalization by signals, including flg22 (18). This serves as a proxy for G protein activation (13). Phosphorylation at the phosphocluster site leads to endocytosis, and thus if the phosphatase responsible for dephosphorylation of these phosphoserines is genetically ablated, we expect that the level of internalization is higher than expected even in the absence of flg22. Therefore, we crossed AtRGS1-YFP into the *atbα-1* loss-of-function mutant and quantitated flg22-induced endocytosis of AtRGS1 (32). We included in our survey three other phosphatases (or subunits) shown to interact with AtRGS1 in a yeast two-hybrid screen (19). These are abscisic acid insensitive 2 (ABI2), type one protein phosphatase 8 (TOPP8), and dual specificity phosphatase 1 (DSP1). ABI2 and TOPP8 are protein phosphatases that cause dephosphorylation events at serine and threonine residues (70). ABI2 has been shown to negatively regulate abscisic acid (ABA) signaling in response to increased ABA (73). TOPP8 is an isozyme in the PP1 phosphatase family, many of which are predicted to act in cell cycle regulation (70). DSP1 is one of many dual-specificity phosphatases in the tyrosine phosphatase (PTP) family (74).

As shown in Figure 5*A*, the basal level of AtRGS1 internalization at time zero was, as previously reported (18), about 20% and loss of the respective phosphatases in *abi2-1*, *dsp1*-*4*, and *topp8-4* mutants had no statistically significant effect (supplemental Fig. S3). However, in the absence of ATBα, the basal level of AtRGS1 was twice that for the other genotypes (*p* < 0.01) and nearly the maximum level of flg22-induced AtRGS1 endocytosis. Likewise, Ser/Thr phosphatase inhibitors, calyculin A (75) and cantharidin (76), accelerated AtRGS1-YFP endocytosis in the absence and presence of flg22 (supplemental Fig. S3). In contrast, AtRGS1-YFP internalization was not affected by treatment with the tyrosine phosphatase inhibitor, sodium orthovanadate (77). Endocytosis is also blocked in the RGS1(3SA)-YFP, which contains three point mutations of Ser residues in the phosphocluster required for AtRGS1 endocytosis: S428A, S435A, and S436A (10). This is consistent with the requisite for phosphorylated Ser/Thr residues in the aforementioned phosphocluster to induce endocytosis (10). Taken together, these observations are consistent with the notion that ATBα is the substrate-recognition subunit of a phosphatase that dephosphorylates AtRGS1 at its phosphoserine cluster.

To further compare the internalization dynamics of AtRGS1-YFP in the presence and absence of ATBα, we utilized endocytosis inhibitors to recapitulate the Col-0 phenotype in *atbα-*1*.* The two major endocytic pathways in plants, clathrin-mediated endocytosis (CME) and sterol-dependent endocytosis (SDE), are associated with AtRGS1-YFP internalization and activation of downstream targets of G-protein signaling (18), including ROS bursts (78, 79). To decrease the basal level of internalized AtRGS1-YFP in *atbα-1*, we incubated seedlings for 2 h with 50 μM tyrphostin A23 (TyrA23) and 5 mM MβCD, which inhibit CME and SDE, respectively (18, 78, 80, 81, 82), resulting in basal level of AtRGS1 internalization similar to Col-0 (Fig. 5, *B* and *C*). This finding is consistent with AtRGS1-YFP phosphorylation driving endocytosis *via* CME and SDE pathways (18). After washing out TyrA23 and MβCD with water, we observed AtRGS1-YFP internalization over a 60-min time course resulting in a return to ∼40% internalized protein, the *atbα-1* basal state of internalization (Fig. 5, *B* and *C*). Additionally, the special pattern of basal AtRGS1-YFP internalization in *atbα-1* is similar to Col-0 treated with flg22 or D-glucose as previously reported (18), with *atbα-1* containing both endosome- (Fig. 5*C*, white arrows) and microdomain-like (Fig. 5*C*, red arrows) internalized structures. D-glucose–induced internalization was 20% higher in *atbα-1* than WT (*p* < 0.01) (supplemental Fig. S3*D*).

### Loss of ATBα Results in AtRGS1-YFP Degradation

**Fig. 6:**
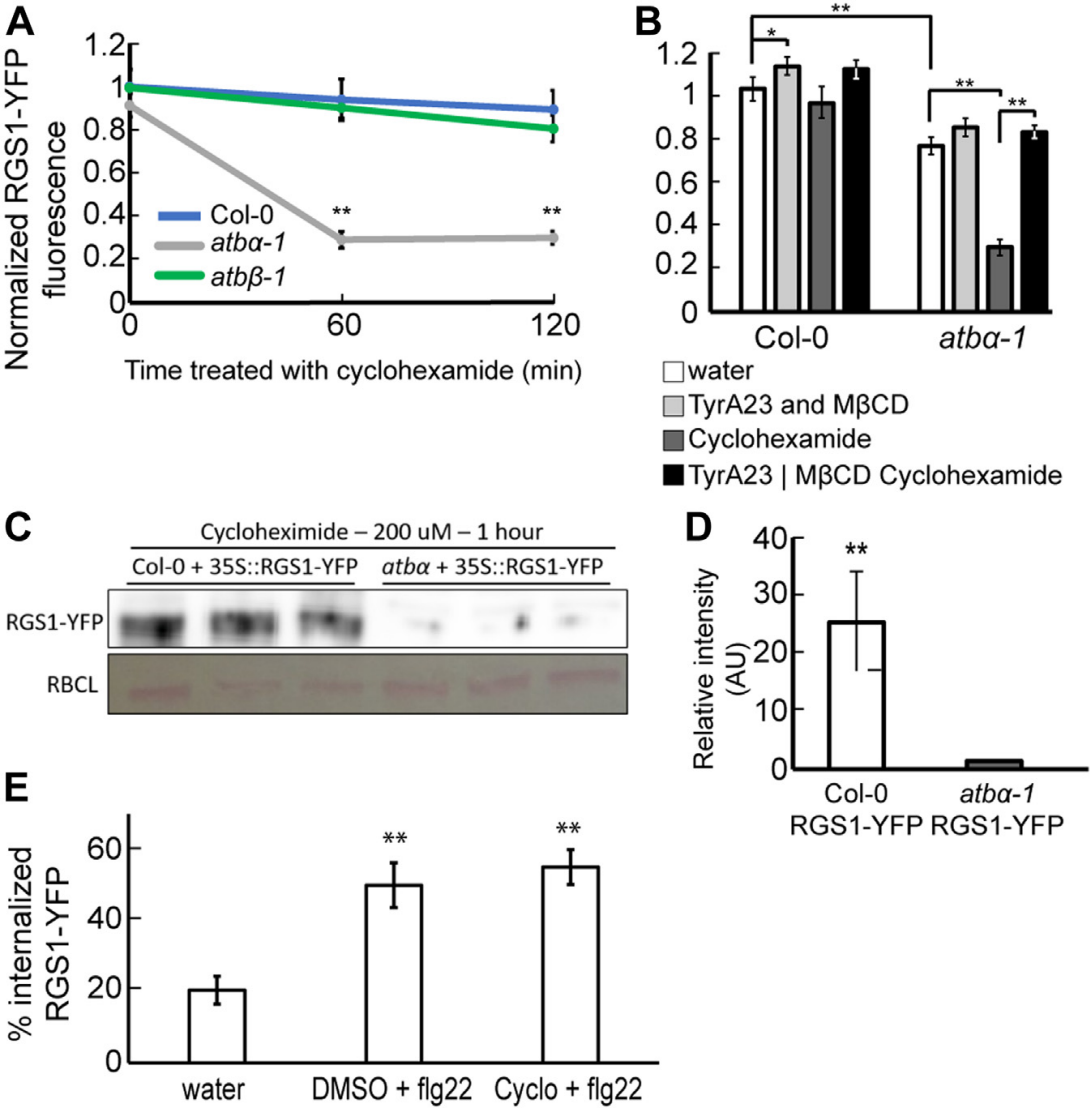
**flg22-induced internalization of RGS1 leads to degradation.***A*, AtRGS1-YFP (noted as RGS1-YFP) protein abundance measured by YFP fluorescence in Col-0, *atbα-1*, and *atbβ-1* treated with 200 μM cycloheximide for 0, 60, and 120 min ∗∗ represents statistical significance (*p* < 0.01) between Col-0 and *atbα-1* within the time point. Error bars represent one confidence interval. n = 25 to 49 across three separate experimental replicates. *B*, RGS1-YFP protein abundance measured by YFP fluorescence in Col-0 and *atbα-1* after treatment with inhibitors. Seedlings were either treated with water, TyrA23 and MβCD, or cycloheximide for 1 h, or they were pretreated with TyrA23 and MβCD for 1 h followed by cycloheximide treatment for 1 h prior to imaging. ∗∗ and ∗ represents statistical significance (*p* < 0.01 or *p* < 0.05 respectively). n = 25 to 49 across three separate experimental replicates. *C*, Western blots of protein extracts from whole seedlings of Col-0 and *atbα-1* expressing RGS1-YFP after treatment with 200 μM cycloheximide for 60 min. The MW of the indicated RGS1-YFP band is ∼80 kDa and RUBISCO Large subunit (RBCL) is ∼56 kDa, *D*, Western blot quantification of AtRGS1-YFP normalized by RuBisCO levels. ∗∗ represents statistical significance (*p* < 0.01) compared to control (RGS1-YFP/Col-0) and determined by unpaired *t* test of three biological replicates. *E*, RGS1-YFP internalization in response to 30 min of flg22 treatment after pretreatment with DMSO or 200 μM cycloheximide for 60 min ∗∗ represents statistical significance (*p* < 0.01) between water and treatment. Error bars represent one confidence interval. n = 25.

Because AtRGS1 phosphorylation is required for internalization and subsequent degradation, the absence of phosphatase activity on this protein is expected to promote lower AtRGS1 levels within the cell. Cycloheximide was used to inhibit protein synthesis in Arabidopsis seedlings treated with flg22 for 1 and 2 h. Confocal microscopy revealed a 70% reduction in AtRGS1-YFP fluorescence levels in *atbα-1* compared to WT (Fig. 6*A*). Additionally, the AtRGS1-YFP reporter in WT plants showed increased protein signal when compared to plants crossed with the *atbα-1* allele, indicating that AtRGS1 levels are naturally lower in the absence of the phosphatase subunit that is responsible for the negative regulation of the internalization process (Fig. 6, *A* and *B*). Pretreatment with the endocytosis inhibitors, TyrA23 and MβCD, recapitulated the WT phenotype in *atbα-1* treated with cycloheximide. This suggests that AtRGS1-YFP internalization results in degradation of the protein and ATBα negatively regulates this process. To further validate this finding, we analyzed AtRGS1-YFP abundance in protein extracts from whole plants *via* immunoblot analysis (Fig. 6, *C* and *D*). We found that cycloheximide treatment decreased AtRGS1-YFP protein abundance in *atbα-1* by nearly 100%. In the presence of the proteasome inhibitor, MG132, the lower level of AtRGS1 in the *atbα-1* mutant was higher (supplemental Fig. S5), suggesting that the degradation of AtRGS1 is proteasome-dependent. The discrepancy of 70% reduction by fluorescence quantitation *versus* 100% by immunoblot quantitation is likely due to the greater dynamic range of confocal microscopy compared to immunoblot quantitation. Nonetheless, in both cases, we observed a vastly reduced steady-state AtRGS1-YFP protein level in *atbα-1*.

Finally, the role of tonic cycling in modulating the percent internalized AtRGS1-YFP in response to flg22 was assessed. To accomplish this, flg22-induced AtRGS1-YFP endocytosis in the presence and absence of cycloheximide was compared. AtRGS1-YFP endocytosis levels were 2.5 and 2.75 times higher in flg22-treated seedlings with and without cycloheximide, respectively, although this was not supported statistically (Fig. 6*E*). This suggests that nascent AtRGS1-YFP is not substantially transported to the membrane during the 30-min flg22 treatment.

### ATBα Modulates the Plant Immune Response and Development

**Fig. 7:**
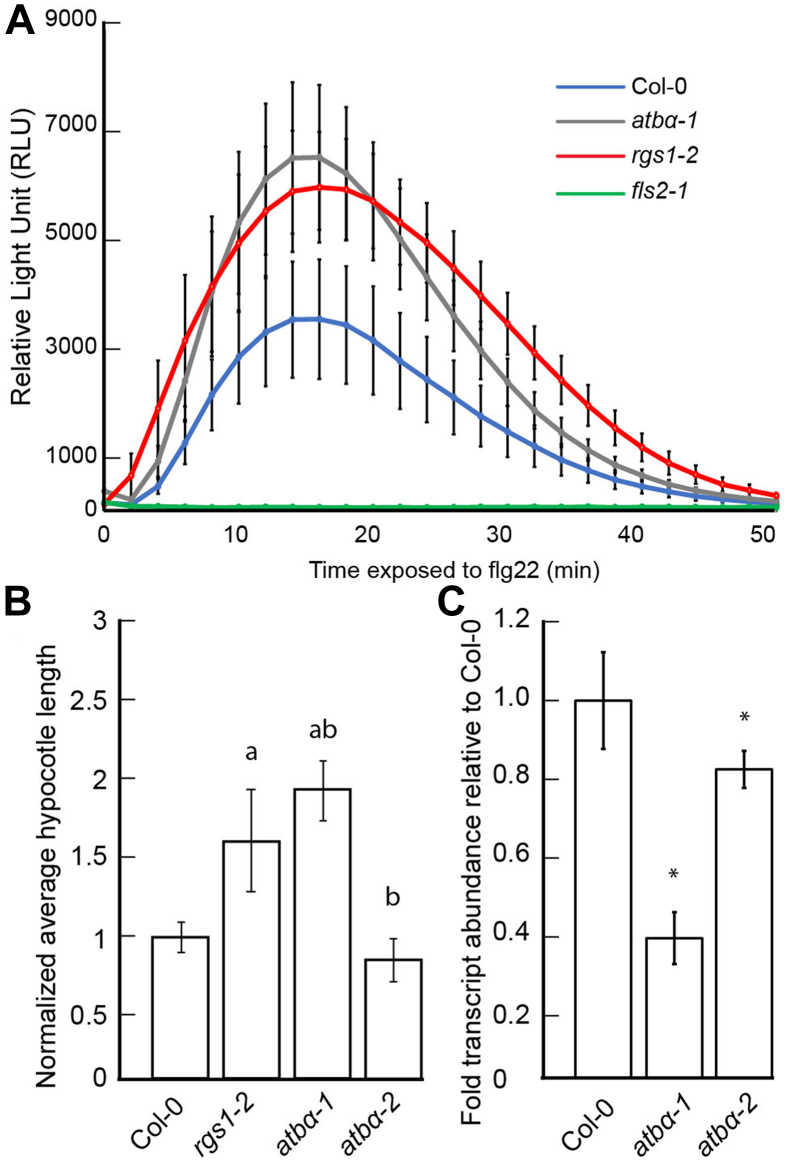
**ATBα modulates plant immune response and development.***A*, flg22-induced ROS, reported as relative luminescence units (RLU), in leaf disks generated from 5-week-old plants treated with 100 nM flg22. Error bars represent one confidence interval. The graph is representative of three separate experiments. n = 20 to 35. *B*, etiolated seedlings were grown for 64 h (25 °C) as described in Experimental Procedures. Germination rate for each genotype is provided in supplemental Fig. S4. Lengths of hypocotyls were obtained as described in Experimental Procedures. Hypocotyl lengths of Col-0, *rgs1-2*, *atbα-1*, and *atbα-2*. (a) denotes significantly difference from Col-0 (*p* < 0.01). (b) denotes significant difference between phosphatase mutant alleles and *rgs1-2* (*p* < 0.01) determined by a two-way ANOVA followed by Tukey’s post hoc test. n = 35 to 50. ∗∗ represents significant difference (*p* < 0.01) between Col-0 and mutants as assessed by ANOVA and Tukey’s posthoc test. *C*, amount of *ATBα* transcript is shown. The relative threshold method Crt-qPCR analysis of the *ATBα* gene in Col-0, *rgs1-2*, *atbα-1*, and *atbα-2* as described in Experimental Procedures. Averages of three biological replicates are reported.

Given that ATBα interacts with and modulates the phosphorylation and subsequent internalization of AtRGS1, we hypothesized that such functions would affect the activation of downstream G-protein signaling targets. One of the most rapid known events in flg22-dependent G protein signaling is the ROS burst, beginning within seconds of recognition of the MAMP and peaking between 10 and 15 min before returning to the base line within 60 min. Because of the involvement of G-protein complexes in flg22-induced ROS production (83, 84), we quantitated flg22-induced ROS production in the *atbα-1* null mutant and compared to *rgs1-2* and Col-0. The peak of ROS production induced by 100 nM flg22 treatment was enhanced in *rgs1-2* compared to WT (Fig. 7*A*), consistent with previous reports (15, 85). The *atbα-1* mutant also showed an enhanced ROS peak compared to WT consistent with increased AtRGS1 internalization and degradation observed in the mutant. A second null allele (supplemental Fig. S6*A*), *atbα*-3, also showed higher flg22-induced ROS (supplemental Fig. S6*B*).

To further characterize differences in the phosphatase mutants related to AtRGS1-dependent G-protein signaling, we measured hypocotyl lengths in etiolated seedlings, a G protein–dependent phenotype (6), and compared them to *rgs1-2*. Etiolated *rgs1-2* seedlings have slightly elongated hypocotyls compared to WT under specific conditions and this is associated with upregulated G protein activity (6). Therefore, if one of these phosphatases modulates G protein signaling, then we would expect changes in embryonic hypocotyl development (57). These established phenotypes were used to reveal potential regulatory effects of phosphatases based on their respective mutant phenotype. Generally, the phosphatase mutant alleles resembled the hypocotyl lengths of *rgs1-2*, which were significantly longer (*p* < 0.01) than Col-0 (Fig. 7*B*). The one exception was *atbα-2*, a weak allele (Fig. 7*C*), which showed a WT phenotype. Specifically, transcriptional analysis showed that all phosphatase mutant alleles showed lower transcript levels than WT, but *atbα-2*, an intron insertion allele, had transcript levels 83% of WT (Fig. 7*C*). The correlation between gene expression of the phosphatase mutant alleles and the hypocotyl elongation phenotype confirm that the phenotype is conferred by loss of ATB*α.*

## Discussion

From the vantage of phosphorylation states, innate immunity in roots is shown here to be rapidly responding and dynamic. While it has been known since the beginning of plant G protein research that G signaling played some role in innate immunity, our systems analysis makes it clear how profound is that role with most of the flg22-regulated phosphosites dependent on a functional G protein complex. Many new avenues to dissect innate immunity are revealed. One of these is a phosphatase that regulates the steady-state phosphorylation status of a key modulator of G signaling. Because the action of this phosphatase leads to a change in G signaling, the second level of dynamics in pattern-triggered immunity is exposed. Specifically, as AtRGS1 levels change with a time constant of minutes, the phosphorylation-dephosphorylation cycle occurring in seconds is predicted to change. A triangular feedback cycle apparently is involved: 1. The G protein complex modulates flg22-induced phosphorylation of a large number of proteins, 2. AtRGS1 modulates the G protein complex activity, and 3. The ATBα phosphatase stabilizes AtRGS1 by reducing its degradation. The exact schematic relationship of these three intertwined regulators requires further analyses of flg22-induced phosphoproteomes in the respective loss- and gain-of-function *atbα* and *rgs1* mutants.

The foundation experiments designed to capture the relevant window of time and physiological concentration of flg22 for our subsequent phosphosite mapping also reveal new information about innate immunity in the root. The spatial and temporal information for flg22-induced signaling was achieved using the fluorescent ROS sensor, DCF (33, 86, 87). Confocal micrographs showed peak fluorescence in the elongation zone (EZ) (Fig. 1*A*), which is consistent with a previous study that demonstrated that the EZ is sensitive to flg22 (88). Additionally, the EZ, but not the root tip, exhibits high gene expression of *FLS2*, the gene encoding the canonical flg22 receptor (89). This is consistent with our results where we did not see significant increases in flg22-induced ROS in root tips, further suggesting that this region is largely insensitive to flg22.

The major role of the heterotrimeric G protein is even clearer in the dataset analysis of well-known components of PAMP recognition and signaling in plants (9). After flg22 recognition by FLS2, which forms an active complex with BAK1, the receptor-like cytoplasmic kinase BIK1 becomes phosphorylated (90, 91). Once phosphorylated, BIK1 triggers a ROS burst response and activates MAPK cascades (92). This dataset shows that the expected differentially-increased levels of phosphorylation from BIK1 in WT, which are decreased in the *quad* mutant and also from the two threonines and tyrosines from each MPK3 (3 and 15 min) and MPK6 (15 min) (93) only occur in the wild type plants and not in the *quad* mutant. This is consistent with the notion that G-protein signaling acts upon upstream, characterized components of plant immunity. At the apex of flg22 signaling lies FLS2/BAK1 which initiates changes in cell behavior (ROS, MAPK cascade, Ca++), some involving BIK1 and others not. flg22-induced phosphorylation of BIK1 *via* BAK1 is attenuated in the absence of the central G protein core and MAPK cascading is eliminated but BIK1 is not required for the latter. This suggests that the plant G-protein complex does not couple the activated receptor to targets (*e.g.* BIK1), rather it is a modulator of the system as a whole. This behavior of the plant G protein mutant has been reported repeatedly in the literature, contrasting sharply with the animal G protein paradigm.

The time course profiles also revealed an additional set of receptors in innate immunity. Among those kinases, some are expected such as wall-associated kinase (94), NSP-interacting kinase 1 (NIK1 (95, 96), chitin elicitor receptor kinase 1 (97), BAK1-related kinase (9), feronia (FER (98), and HAESA (99), while 22 other kinases present opportunities. Changes in the phosphorylation of kinases in innate immunity were analyzed and shown in supplemental Table S4. All RLKs identified among the phosphorylated proteins are provided in Supplemental Dataset 4.

Our results were refined with data from the Arabidopsis experimental protein-protein interaction network, the Arabidopsis Immune Network, and the Arabidopsis G Protein Interactome database (AGIdb). The AGIdb was generated in a previous study by investigating interactions among proteins involved in the G protein pathway, using a yeast-two-hybrid approach combined with bimolecular fluorescence complementation (19). This interactome contains 4 Ser/Thr phosphatases that interact with AtRGS1, including ATBα, ABI2, TOPP8, and DSP1. Of the four phosphatases, only ATBα showed flg22-induced, G protein-independent phosphorylation (Fig. 3*G*).

We examined the overlap between our results and several previous studies that measured elicitor-induced phosphoproteome changes. We used three published flg22-induced phosphoproteome datasets that each utilized different treatment times and tissues. We also compared these results with a xylanase-, and oligo-galacturonide- (pectin fragments, OG) induced phosphoproteome, respectively (90, 100, 101, 102). The comparison required that a phosphoprotein contain differentially-expressed phosphosites(s) in at least one other published dataset. This resulted in 185 phosphoproteins that were present in two or more datasets despite major differences in experimental design and lesser proteome coverage of the earlier technology. Of the 185 proteins, only 12 (∼5%) have phosphosites that are differentially expressed in the *quad* mutant at 3- or 15-min post flg22.

In particular, one study focused on changes in phosphorylation in response to OGs (100). A 5-min treatment was sufficient to induce phosphorylation of 50 different proteins and, of this set, ATBα was phosphorylated at two serine residues, 467 and 470. Both of which are reduced by flg22 in WT, suggesting an overlapping role of ATBα in the OG and flg22 response pathways.

Among the genes present in numerous datasets, the protein encoded by open stomata 2 (*OST2*) was found to be phosphorylated in response to flg22 in two datasets. OST2 is integral to induce stomatal closure in response to ABA (103), which is important for plant growth in response to drought as well as bacterial invasion. Our results suggest that OST2 is jointly regulated *via* ABA and flg22 to illicit stomatal closure *via* two different stimuli. Another protein of interest across studies is FER. FER phosphorylation was altered in three out of four flg22 phosphoproteomes. In guard cells, FER interacts directly with the Gβγ dimer of the heterotrimeric G protein complex (104). Other notable genes found in overlapping phosphoproteomes are genes related to MAPK and Ca^2+^ signaling, including MAPK4 and 6 and CPK5, 9, and 13.

Because the vast majority of flg22-induced phosphosites depend on a functional G protein complex, one may argue that G signaling is at the apex of this pathway but the effect of G proteins in flg22-induced changes in the phosphoproteome could also be indirect. There are many possibilities. For one, the receptor, FLS2, may not be trafficked to the plasma membrane in the *quad* mutant. This does not exclude many other possibilities: post-translational modifications of FLS2 such as phosphorylation, ubiquitination, and SUMOylation, the lack of a co-receptor to FLS2, modification of another protein in the receptor complex, and interaction with a partner are possibilities to name just a few. BIK1 acts immediately downstream of flg22-bound FLS2. Therefore, still another plausible mechanism for the major differences in the flg22-induced phosphoproteomes between WT and the G protein *quad* mutant is that the BIK1 steady-state level or its activity is altered in the mutant. Indeed, we show that BIK1 abundance as measured by MS-MS is reduced in the *quad* mutant (supplemental Dataset S2) thus this plausible mechanism is currently the leading hypothesis to be tested. It should be noted that no plant signal has yet been shown to be directly coupled from a plasma membrane receptor by the heterotrimeric G protein and that the plant G protein complex acts more likely as a modulator of signaling rather than a direct coupler (105). This state of our understanding is consistent with the above hypothesis.

Given that plant pathogen effectors target the highly-connected hub proteins preferentially over other proteins of the interactome (26, 28, 29, 49), we investigated the 3734 phosphoproteins interactions in Arabidopsis experimental PPI (PPIE) network (19, 43, 49, 106). The PPIE contains 257 G-proteins interactome nodes, 235 immune network nodes, 111 effector nodes and other proteins. Pathogen effectors target the highly connected nodes more efficiently to hijack the host system, the next line of investigation was to perform network topology analysis, specifically degree centrality to calculate the total interactions (25). We show that the phosphorylated proteins possess significantly higher degree distribution than the non-phosphorylated proteins (Fig. 3*B*; Student’s *t* test *p*-value ≤0.001) suggesting the potential roles of highly connected nodes in efficacious signal transduction in response to pathogens or pathogen-mimic stimuli. As a result, we found 352 proteins as hub_15_ (nodes with ≥15 interactions) of which 278 are phosphorylated in our analysis. Further, we expanded our analysis of hub_15_ in the Arabidopsis immune network and G-protein interactome nodes, and report that irrespective of the group, the phosphorylated hubs are significantly over-enriched as compared to unphosphorylated hubs which are under-enriched in PPIE, immune and G-protein interactome nodes (Fig. 3*C*, hypergeometric test *p*-value ≤0.001). Collectively, these results further advance the importance of G-proteins as highly connected signal transducer that host proteins and potential pathogen targets in plant immune network. Overall, our network analysis also corroborates with the findings that the majority of proteins in the flg22-induced proteome is regulated by G-proteins.

As such, we compared phosphoproteins in our dataset to the experimental interactome and immune network (Fig. 3*A*). This highlighted a set of targets of pathogen effectors, suggesting the involvement of these genes in the flg22-induced phosphoproteome and the pathogen's targeting of them to suppress MAMP-triggered immunity. Among the connections between genes in our dataset and these two interactomes, we discovered the same interactions of G proteins and effectors, including PP2A-A2, a structural subunit of PP2A. PP2A is comprised of 3 structural subunits: A1, A2, and A3 (70). We found that A2 was phosphorylated after 15 min of flg22 treatment, and that phosphorylation levels were reduced in the *quad* mutant, suggesting these phosphosites are dependent on the G-protein. Interestingly, A2 is linked to plant defense pathways (107). A2 also has a role in mitigating vesical trafficking *via* PIN proteins (108, 109) and is involved in redox signaling in peroxisomes (110). When considering the heterotrimeric nature of PP2A together with the diverse pathways that the A and B subunits of PP2A are associated with, it is possible that the PP2A phosphatase regulate numerous parts of the flg22 pathway *via* different arrangements of subunits. Future studies should examine which structural and catalytic subunits interact with ATBα to regulate flg22-induced endocytosis.

Taken together, the physical (Fig. 4) (19) biochemical (Fig. 4), bioinformatic (Fig. 3*E*), and genetic interaction (Figs. 5 and 7) data place one of the dynamically phosphorylated targets, ATBα, into a G-protein-dependent plant immunity network and ends the long search for the AtRGS1 phosphatase. This new information is important because Arabidopsis encodes 17 substrate-specificity B subunits of PP2A, which interact in a variety of combinations to yield differentially-regulated outcomes (111, 112) and are broken down into 3 subfamilies. ATBα and ATBβ, make up their own subfamily characterized by 5 similar WD40 repeats that are unlike all other isoforms of the B subunit (70). B subunits of PP2A are directly related to stress signaling (112), and root growth (113). PP2A is also a negative regulator of the PAMP-triggered immune response in Arabidopsis (114). Here, the PP2A holoenzyme composed of A1 and B’η/ζ was found to inhibit PAMP-triggered immune responses *via* association with BAK1, a key immune component of the flg22-signaling pathway (14, 115). Genetic ablation of some B subunits led to increased steady-state BAK1 phosphorylation and flg22-induced ROS bursts.

To our knowledge, PP2A (ATBα type) is the first phosphatase associated with any 7-transmembrane GPCR-like protein. One other PP2A (B56δ type) was discovered by a phosphoproteomic screen to be activated by cAMP and may be important in GPCR-based signaling but it is not known if this phosphatase dephosphorylates a GPCR (116). Both B56δ and ATBα are B subunits as described above, however, ATBα has a 5-bladed WD40-repeat scaffold while B56δ has a B56 scaffold. Nonetheless, our study prompts a set of experiments for animal G signaling mechanisms.

In conclusion, our study provides a novel role for ATBα and the heterotrimeric PP2A phosphatase in regulating flg22-induced, G protein-dependent signaling. We also showed that the ATBβ isomer does not share this functionality despite being in the same subfamily with ATBα and containing a similar peptide sequence. Furthermore, our phosphoproteome dataset provides a detailed account of the G protein-dependent phosphorylation events that occur early in the flg22 signaling pathway.

## Data Availability

Raw proteomics data including peaks and spectra have been deposited on MassIVE with accession number MSV000092672 (ftp://massive.ucsd.edu/MSV000092672/).

FTP for reviewers: ftp://MSV000092672@massive.ucsd.edu

Password: “watkins_flg22”

## Supplemental data

This article contains supplemental data.
