# Supplementary material for: Phosphorylation Dynamics in a flg22-Induced, G Protein–Dependent Network Reveals the AtRGS1 Phosphatase
Source: Mol Cell Proteomics. 2023 Dec 20;23(2):100705. doi: 10.1016/j.mcpro.2023.100705 (PMC10837098; doi:10.1016/j.mcpro.2023.100705)
Supplement: Supplemental Tables and figures [file mmc2.pdf]

SUPPLEMENTAL INFORMATION

Table of contents

Table S1: Phosphatase mutant primer sequences

Table S2: Published phosphoproteomes method overview

Table S3: TMT labeling strategy

Table S4: Summary of phosphorylation changes on critical kinases

Figure S1. Volcano plots of differential protein abundance and phosphosite expression.

Figure S2. GST Control: The level of RGS1 phosphorylation was not changed by adding a different GST protein, GST-RACK1.

Figure S3. RGS1-YFP internalization in response to phosphatase inhibitors and phosphatase null mutations.

Figure S4. Germination rates of the genotypes tested.

Figure S5. The steady-state level of RGS1 is lower in the *atbα1* mutant in a proteosome-dependent manner.

Figure S6. A second null allele of ATBα, *atbα*-3, has greater flg22-induced burst than wild type. Genotypes of two other *atbα* mutants provided, *atbα*-5 and *atbα*-6.

Excel Spreadsheets labeled Datasets S1-S4 are provided separately

Supplemental Dataset S1: Phosphosites

Supplemental Dataset S2: Protein Abundance

Supplemental Dataset S3: flg22 induced protein-protein interaction experimental (PPIE)

Supplemental Dataset S4: All receptor-like kinases (RLKs)

Table S1: Phosphatase mutant primer sequences

Mutant Allele (Phosphatase Gene)	Genotyping		Left Border (LB) Primer of T-DNA Insertion	qPCR	
	LP	RP		LP	RP
SALK_015166C (ABI2)	N/A	N/A	SALK LBb1.3: ATTTTGCCGATTTCGGAAC	GAACGGGGCTCGTGTATTTGGT	TTGCTGCAGGATGTTTTCCTTCTCC
SAIL_547_C10 (ABI2)	TTCCTTCTCCTCTTTTCTCCG	TTGATCCGAGATCGATGAATC	SAIL LB1: GCCTTTTCAGAAATGGATAAATAGCCTTGCTTCC	GAACGGGGCTCGTGTATTTGGT	TTGCTGCAGGATGTTTTCCTTCTCC
SALK_032080C (ATBα)	N/A	N/A	SALK LBb1.3	CCAGAGCAAGCAGGTCCTAAATCGT	GCTCGCAAGCCATTGCCACTTATAC
SALK_0950040C (ATBα)	N/A	N/A	SALK LBb1.3	CCAGAGCAAGCAGGTCCTAAATCGT	GCTCGCAAGCCATTGCCACTTATAC
SALK_125184 (TOPP8)	N/A	N/A	SALK LBb1.3	CGCTGGTGCGTTATTAAGCGTTG	GCTTGAGCTGTGGAACCGTGATATT
SALK_076144 (TOPP8)	ATTGCAATAGTGCTCCCACTG	TGCTTTAACGCTCGTCAAATC	SALK LBb1.3	CGCTGGTGCGTTATTAAGCGTTG	GCTTGAGCTGTGGAACCGTGATATT
WiscDsLox473B10 (DSP1)	TTGTTTTGCAAAACTGCAAAG	TTGCCTTCAATACCAAACTGG	WISCDSLOX: AACGTCCGCAATGTGTTATTAAGTTGTC	CCGGCGAAGAACTTCACCTAATTC	TCAGTGCCATGCGGATTTTATGG
SAIL_116_C12 (DSP1)	TTTGTTTTGCAAAACTGC	GTTTGGTATTGAA	SAIL LB1	CCGGCGAAGAACTTCACCTAATTC	TCAGTGCCATGCGGATTTTATGG
atbα-3: salk_027428	tcgaacaatcaaagaacaccc	aaagccaatttacagctaagcag	SALK LBb1.3	CTCTGCAACTAAACTGCTACC	TCCGTTTTTTTGCTCTCTTGTGC
atbα-5 : Wiscseq_DsLox340A05.0(CS851840)	GCCAGCAGGAGGCATCTCTTC	GCTGATGTGATAACCTctgcatg	TCCTCGAGTTTCTCCATAATAATGT	CGAAGAGTATATGCTCATGCTC	CCATTTTCGCAGGCTTGACATC
atbα-6: Wiscseq_DsLoxHs084_08E.1 (CS908029	GCCAGCAGGAGGCATCTCTTC	GCTGATGTGATAACCTctgcatg	TGATCCATGTAGATTTCCCGGACATGAAG	CGAAGAGTATATGCTCATGCTC	CCATTTTCGCAGGCTTGACATC

Table S2: Published phosphoproteomes method overview

	Benschop, JJ. 2007	Nühse, TS. 2007	Rayapuram, N. 2014	Kohorn, BD. 2016	Watkins, JM. 2021 (and this study)
**Plant tissue**	Cultured cells	Cultured cells	Seedlings	Seedlings	12-day-old roots
**Stimulus**	1000 nM flg22 or 100 µg/ml xylanase	100 nM flg22	1000 nM flg22	50 µg/ml OG	50 nM flg22
**Time treated**	10 min	3-15 min	15 min	5 min	3 and 15 min

Benschop JJ, Mohammed S, O'Flaherty M, Heck AJR, Slijper M & Menke FLH (2007) Quantitative phosphoproteomics of early elicitor signaling in Arabidopsis. *Mol Cell Proteomics MCP* 6: 1198–1214

Nühse TS, Bottrill AR, Jones AME & Peck SC (2007) Quantitative phosphoproteomic analysis of plasma membrane proteins reveals regulatory mechanisms of plant innate immune responses. *Plant J Cell Mol Biol* 51: 931–940

Rayapuram N, Bonhomme L, Bigeard J, Haddadou K, Przybylski C, Hirt H & Pflieger D (2014) Identification of novel PAMP-triggered phosphorylation and dephosphorylation events in *Arabidopsis thaliana* by quantitative phosphoproteomic analysis. *J Proteome Res* 13: 2137–2151

Kohorn BD, Hoon D, Minkoff BB, Sussman MR & Kohorn SL (2016) Rapid Oligo-Galacturonide Induced Changes in protein phosphorylation in Arabidopsis. *Mol Cell Proteomics MCP* 15: 1351–1359

Watkins JM, Ross-Elliott TJ, Shan X, Lou F, Dreyer B, Tunc-Ozdemir M, Jia H, Yang J, Oliveira CC, Wu L, *et al* (2021) Differential regulation of G protein signaling in Arabidopsis through two distinct pathways that internalize AtRGS1. *Sci Signal* 14: eabe4090

Table S3: TMT Labeling Strategy

	3 min sample run	15 min sample run
TMT_1	H2O_3min_WT_R1	H2O_15min_WT_R1
TMT_2	H2O_3min_WT_R2	H2O_15min_WT_R2
TMT_3	H2O_3min_WT_R3	H2O_15min_WT_R3
TMT_4	flg22_3min_WT_R1	flg22_15min_WT_R1
TMT_5	flg22_3min_WT_R2	flg22_15min_WT_R2
TMT_6	flg22_3min_WT_R3	flg22_15min_WT_R3
TMT_7	H2O_3min_quad_R1	H2O_15min_quad_R1
TMT_8	H2O_3min_quad_R2	H2O_15min_quad_R2
TMT_9	H2O_3min_quad_R3	H2O_15min_quad_R3
TMT_10	flg22_3min_quad_R1	flg22_15min_quad_R1
TMT_11	flg22_3min_quad_R2	flg22_15min_quad_R2
TMT_12	flg22_3min_quad_R3	flg22_15min_quad_R3
TMT_13	0minWT_R1	0min-quad_R1
TMT_14	0minWT_R2	0min-quad_R2
TMT_15	0minWT_R3	0min-quad_R3
TMT_16	Pooled Ref	Pooled Ref

Table S4: Summary of phosphorylation changes on critical kinases

Kinase	Note	Change at 3 min	Change at 15 min
BAK1 (AT4G33430.1)	Detected phosphorylation on AA 290, 381, 612, 443, 446, 449, 450, 455.<br>Note that some of the phoshopeptides do not distinguish between BAK1 and SERK1 and thus these potential BAK1 IDs are listed under ATSERK1.<br><br>Protein abundance quantified. No change in BAK1protein level		Triple phosphorylated 443/446/449 has increased phosphorylation in the quad mutant at 15 min. No change in WT in response to flg22.<br><br>Singly phosphorylated 449 has decreased phosphorylation in quad mutant at 15 min. No change in WT.<br><br>Singly phosphorylated 455 has decreased phosphorylation in quad mutant at 15 min. No change in WT.
BIK1 (AT2G39660)	Detected phosphorylation on AA 19 and 28<br><br>Protein abundance quantified at 15 min. BIK1 protein level decreases.	Double phosphorylated form<br>is increased in WT flg22 at 3 min (q-value 0.03; FC 1.23)<br><br>Is decreased in quad at 3 min based on old cutoff (q-value 0.09; FC -1.3)	Double phosphorylated form is increased in WT flg22 at 15 min (q-value 0.04; FC 1.2)<br><br>is decreased in quad following flg22 at 15 min (q-value 0.0007; FC -1.5)
CDPK5 (AT4G35310.1)	Detected phosphorylation on AAs 33, 34, 36, 39, 544, 548, 552<br><br>Protein abundance quantified. No change in WT. CDPK5 protein is decreased in quad mutant following 3 and 15 of flg22 treatment	Lots of DE<br>-<br>General pattern is decreased levels of phosphorylation in both WT and quad mutant after flg22 at 3 min	At 15 min 33, 34, 36, 39 phosphorylation is not changed in WT in response to flg22. However, phosphorylation is decreased on these sites in the quad mutant
MPK3 (AT3G45640.1)	Phosphorylated at 196, 198. This is the TEY motif in activation loop.	Double phosphorylated (i.e. active form):<br><br>increased in WT 3 min flg (q=0.03; FC 1.2)<br><br>decrease in quad at 3 min post flg (q=0.03; FC -1.47)	Double phosphorylated (i.e. active form):<br><br>increased in WT 15 min flg (q=0.03; FC 2.2)<br><br>decrease in quad at 15 min post flg (q=0.0; FC -2.3)
MPK6 (AT2G43790.1)	Phosphorylated at 221, 223. This is the TEY motif in activation loop.<br><br>Protein abundance quantified. MPK6 protein level decreases in WT and quad at 3 min flg22. Also decreased in quad at 15 min post flg22	Double phosphorylated (i.e. active form):<br><br>No changes at 3 min	Double phosphorylated (i.e. active form):<br><br>increased in WT 15 min flg (q=0.03; FC 1.3)<br><br>decrease in quad at 15 min post flg (q=0.001; FC -1.4)

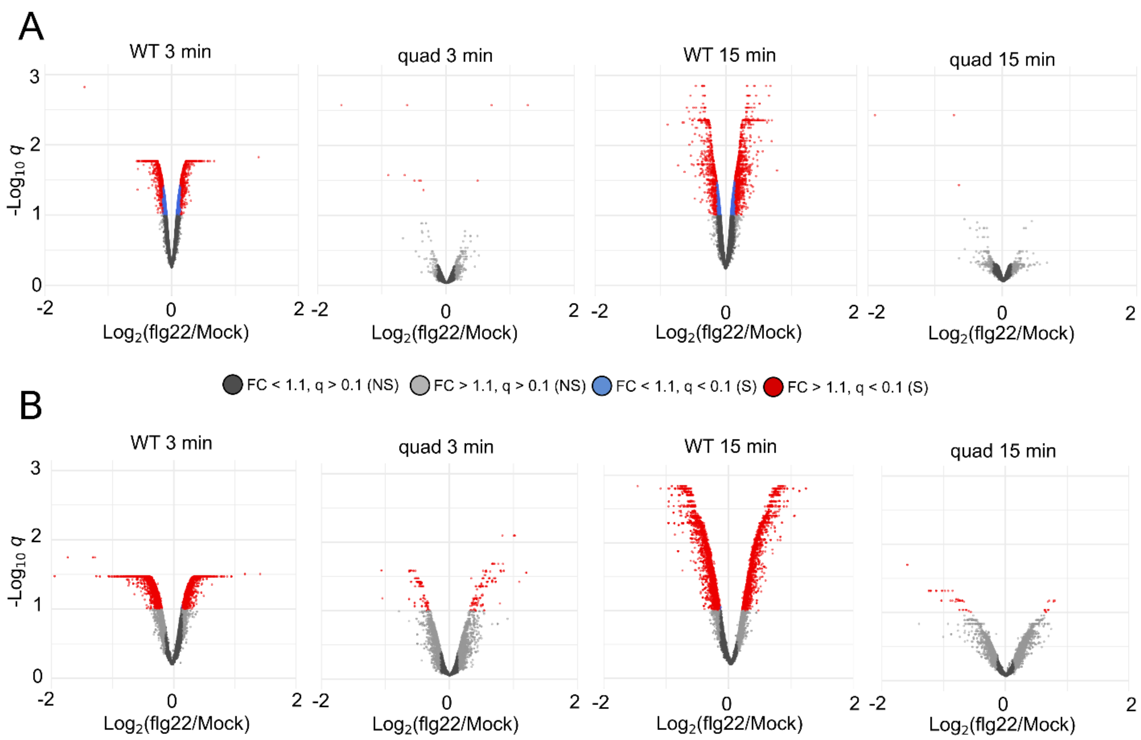

Figure S1. Volcano plots of differential protein abundance and phosphosite expression.

A. Differential protein abundance
B. Differential phosphosite response to flg22 treatment in WT and quad mutants after 3 or 15 min. Each dot represents one protein group (n = 8,918) or phosphosite (n = 24,468). x-axis is log2(flg22/mock). y-axis is -log10(q-value). Protein groups/phosphosites with a significant q-value (q < 0.1) are in blue (fold-change < 1.1) and red (fold change > 1.1). Protein groups/phosphosites with a non-significant q-value (q > 0.1) are in gray. S – significant, NS, not significant

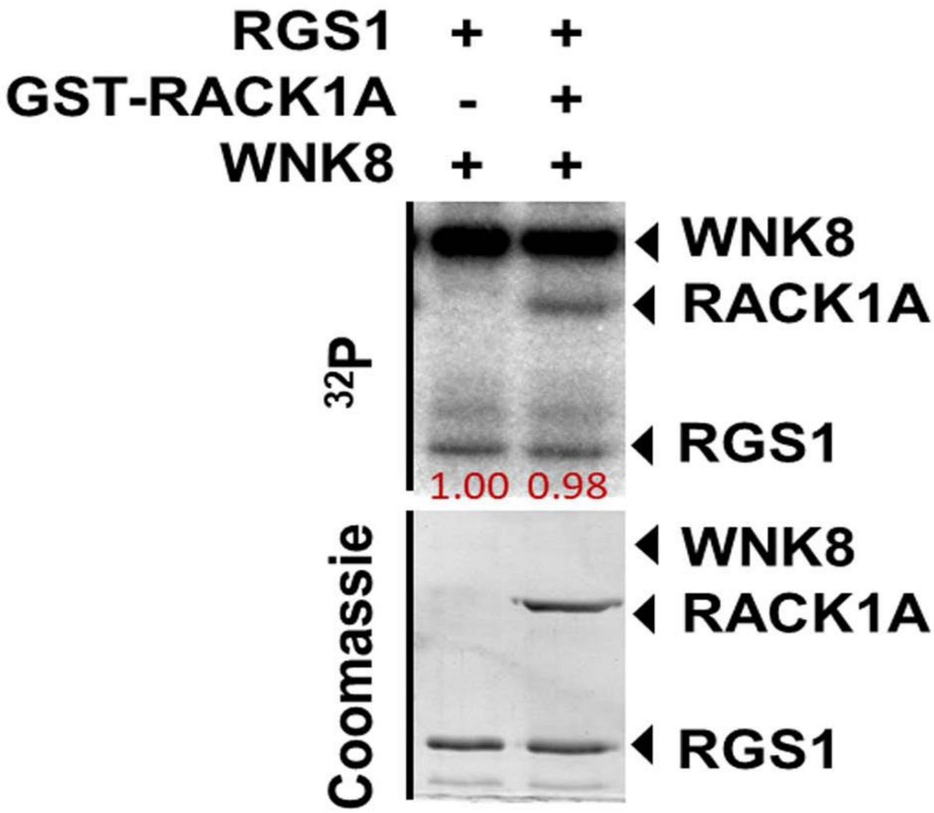

Figure S2. GST Control: The level of RGS1 phosphorylation was not changed by adding a different GST protein, GST-RACK1. This control experiment was performed as described in Materials and Methods in parallel to the experiment described in Figure 4. RACK1A is GST tagged. The kinase WNK8 and AtRGS1 (RGS1) are tagged with 6X poly-His.

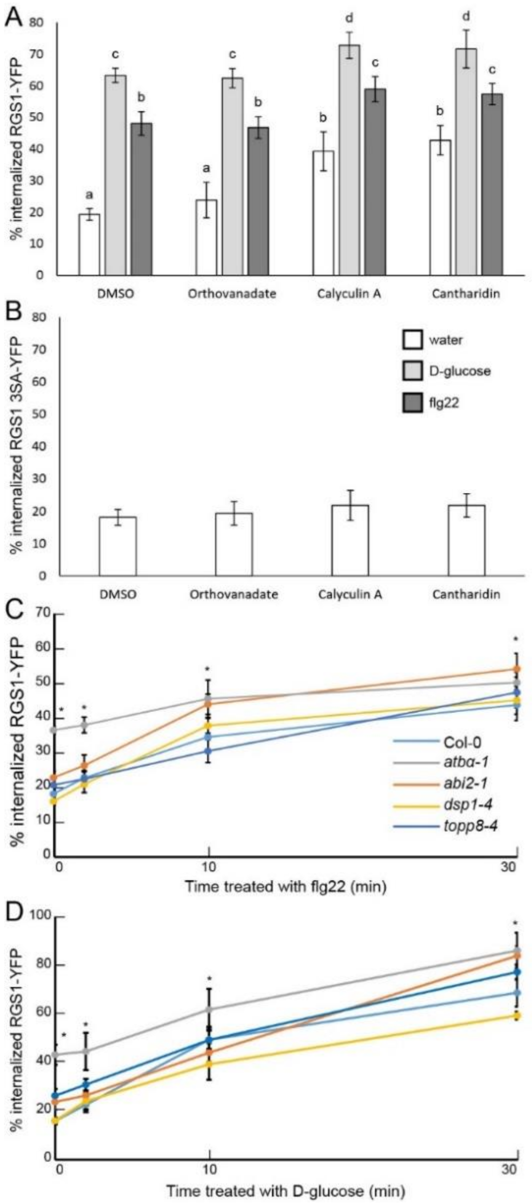

Figure S3. RGS1-YFP internalization in response to phosphatase inhibitors and phosphatase null mutations.

A. D-glucose- or flg22-induced RGS1-YFP internalization after pretreatment with DMSO or phosphatase inhibitor: orthovanadate, calyculin A, and cantharidin for 2 hours. Means with different letters indicate significant difference ($P < 0.05$). Error bars represent CI. n = 15-30.

B. RGS1-YFP internalization after treatment with DMSO or phosphatase inhibitor for 2 hours. Error bars represent CI. n = 28-35.

C. flg22-induced RGS1-YFP internalization measured over time in Col-0 and null phosphatase mutants. * Represents statistical significance ($P < 0.01$) between Col-0 and *atbα*-1. Error bars represent CI. n=30 across three separate experimental replicates.

D. D-glucose-induced RGS1-YFP internalization measured over time in Col-0 and null phosphatase mutants. * Represents statistical significance ($P < 0.01$) between Col-0 and atbα-1. Error bars represent CI. n=27-35 across three separate experimental replicates.

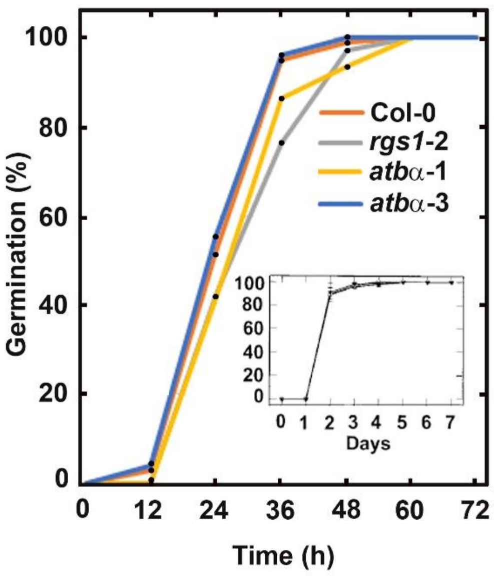

Figure S4. Germination rate of the tested genotypes. Matched seeds of the indicated genotypes were sterilized, stratified, and plated on 1/2X MS media 1% sucrose as described by Ullah, et al (2002) except that the stratification was for 6 days and the germination and growth temperature was 25°C. Germination was scored positive if a radical was observed microscopically. Each genotype is indicated by the color of the line. Inset: Figure from Ullah H, Chen J-G, Wang S, & Jones AM (2002) Role of a heterotrimeric G protein in regulation of Arabidopsis seed germination. *Plant Physiology* **129**:879-907 modified with permission. Symbols: Ws-1 wildtype ecotype (open circles), *gpa1*-1 (black circles), *gpa1*-2 (black inverted triangles. Seedlings are under the same conditions as described in Materials and Methods except stratification was for 4 days.

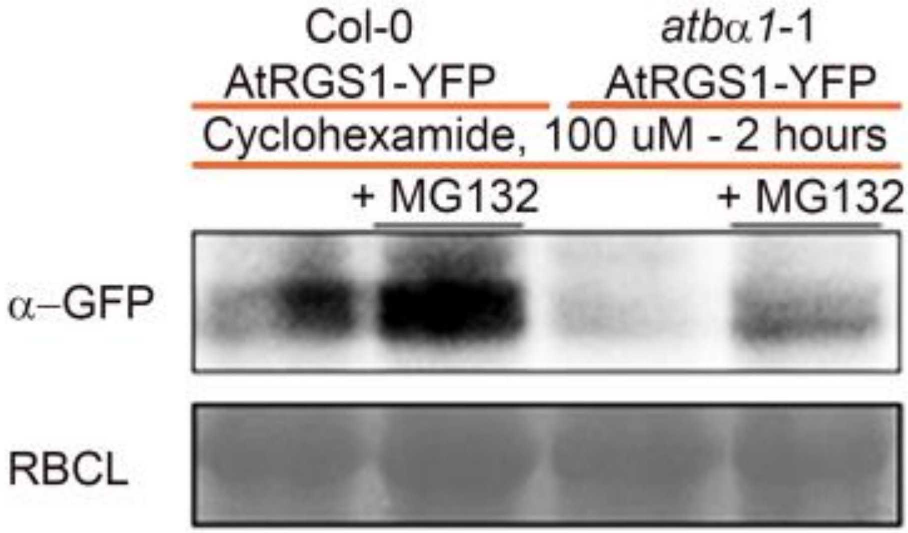

Figure S5. The steady-state level of RGS1 is lower in the *atbα1* mutant in a proteosome-dependent manner. Tissue from Col-0 and *atb1*-1 were prepared as described in the Materials and Methods. The level of RUBISCO (RBCL) is used as the loading control. The blot was probed with anti GFP (α-GFP) to detect AtRGS-YFP. The lower level of AtRGS1-YFP increases with the addition of the proteasome inhibitor, MG132. This experiment was repeated once.

A

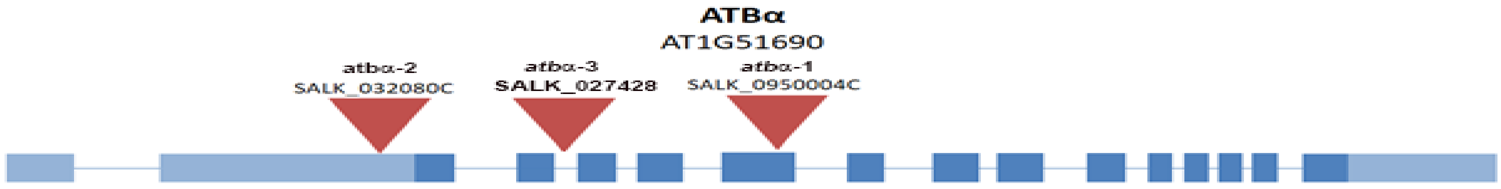

B

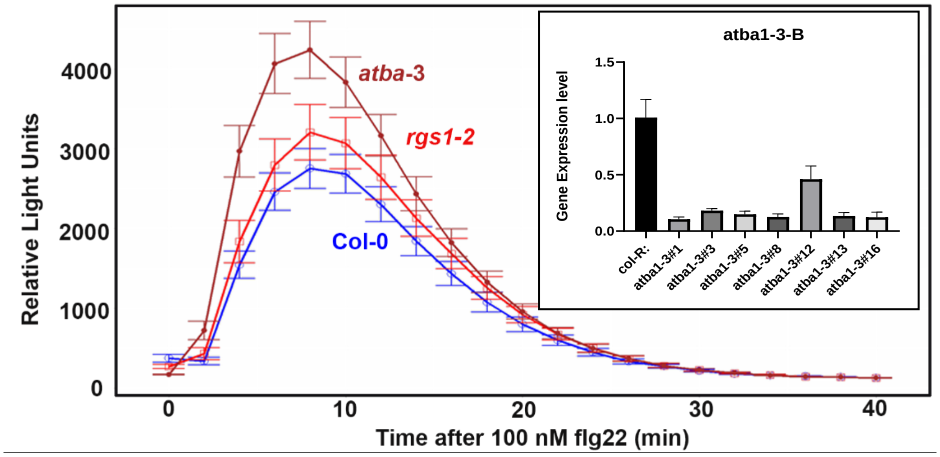

C.

AT1G51690.3 Additional ATBα mutants for this study:

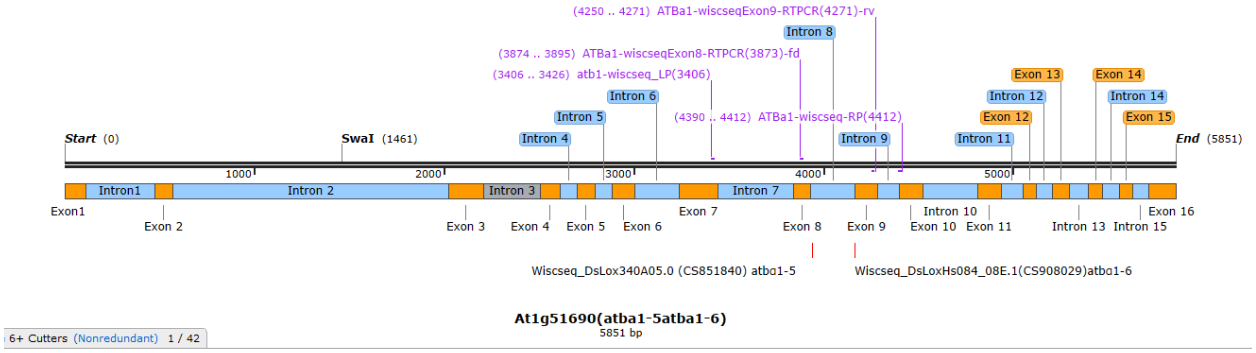

Mutants:

Wiscseq_DsLoxHs084_08E.1 (CS908029) **atbα-6

Wiscseq_DsLox340A05.0 (CS851840) **atbα-5

D.

Primers:

ATBa1-wiscseq_LP(3406): GCCAGCAGGAGGCATCTCTTC
ATBa1-wiscseq-RP(4412): GCTGATGTGATAACCTctgcatg

WiscDs LB TCCTCGAGTTTCTCCATAATAATGT

WiscDsLoxHs L4 TGATCCATGTAGATTTCCCGGACATGAAG

ATBa1-wiscseqExon8-RTPCR(3873)-fd: CGAAGAGTATATGCTCATGCTC

ATBa1-wiscseqExon9-RTPCR(4271)-rv:CCATTTTCGCAGGCTTGACATC

E.

PCR result: qPCR result of individual atbα-5 & -6 alleles:

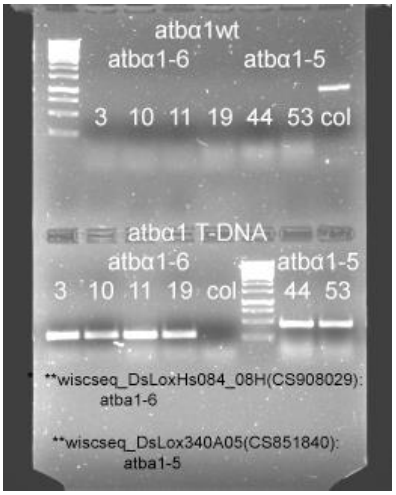

F.

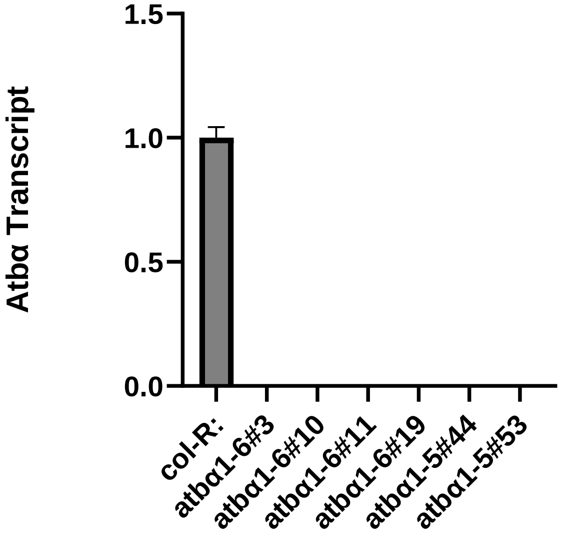

Figure S6. A second null allele of ATBα has greater flg22-induced burst than wild type. **A.** Positions of the T-DNA insertion in the *atbα* mutants used in this study. **B**. flag-induced ROS burst in Col-0, *rgs1*-2 and *atbα*-3 null mutants performed as described in Material and Methods. Error bars are standard deviation. This experiment was repeated 5 times. **Inset**. Expression level of *ATB*α in the *atbα*-3 mutant. Col-0 wild type control is black bars. Col-R is wildtype Col-0; atb1-3 #1, #5, #8, #12, #13, and #16 are individuals of the *atb*α-3 mutant. **C.** Additional *atb*α mutant alleles generated. *atb*α-5 and -6 alleles shown with insertion positions indicated. **D**. Genotyping primers shown. **E**. Genotyping gel using primers in D. **F.** Transcript levels of indicated genotypes. Col-R is wildtype Col-0; atb1-6 #3, #10, #11, and #19 are individuals of the *atb*α-6 mutant; atb1-5 #44 and #53, are individuals of the *atb*α-5 mutant. All mutants shown in this Figure were deposited into the Arabidopsis Biological Resources Center. Please cite this paper when you used.
